# β‐arrestin 2 negatively regulates lung cancer progression by inhibiting the TRAF6 signaling axis for NF-κB activation and autophagy induced by TLR3 and TLR4

**DOI:** 10.1038/s41419-023-05945-3

**Published:** 2023-07-13

**Authors:** Ji Young Kim, Ji Hye Shin, Mi-Jeong Kim, Yeeun Kang, Ji Su Lee, Juhee Son, Soo-Kyung Jeong, Daesik Kim, Duk-Hwan Kim, Eunyoung Chun, Ki-Young Lee

**Affiliations:** 1 Department of Immunology, Samsung Biomedical Research Institute, Sungkyunkwan University School of Medicine, Suwon, 16419 Republic of Korea; 2 R&D Center, CHA Vaccine Institute, Seongnam-si, 13493 Republic of Korea; 3 Department of Precision Medicine, Sungkyunkwan University School of Medicine, Suwon, 16419 Republic of Korea; 4 Department of Molecular Cell Biology, Sungkyunkwan University School of Medicine, Suwon, 16419 Republic of Korea; 5 Department of Health Sciences and Technology, Samsung Advanced Institute for Health Sciences & Technology, Samsung Medical Center, Sungkyunkwan University, Seoul, 06351 Republic of Korea; 6 Single Cell Network Research Center, Sungkyunkwan University School of Medicine, Suwon, 16419 Republic of Korea

**Keywords:** Cancer genomics, Non-small-cell lung cancer

## Abstract

β‐arrestin 2 (ARRB2) is functionally implicated in cancer progression via various signaling pathways. However, its role in lung cancer remains unclear. To obtain clinical insight on its function in lung cancer, microarray data from lung tumor tissues (LTTs) and matched lung normal tissues (mLNTs) of primary non-small cell lung cancer (NSCLC) patients (*n* = 37) were utilized. ARRB2 expression levels were markedly decreased in all 37 LTTs compared to those in matched LNTs of NSCLC patients. They were significantly co-related to enrichment gene sets associated with oncogenic and cancer genes. Importantly, Gene Set Enrichment Analysis (GSEA) between three LTTs with highly down-regulated ARRB2 and three LTTs with lowly down-regulated ARRB2 revealed significant enrichments related to toll-like receptor (TLR) signaling and autophagy genes in three LTTs with highly down-regulated ARRB2, suggesting that ARRB2 was negatively involved in TLR-mediated signals for autophagy induction in lung cancer. Biochemical studies for elucidating the molecular mechanism revealed that ARRB2 interacted with TNF receptor-associated factor 6 (TRAF6) and Beclin 1 (BECN1), thereby inhibiting the ubiquitination of TRAF6-TAB2 to activate NF-κB and TRAF6-BECN1 for autophagy stimulated by TLR3 and TLR4, suggesting that ARRB2 could inhibit the TRAF6-TAB2 signaling axis for NF-κB activation and TRAF6-BECN1 signaling axis for autophagy in response to TLR3 and TLR4. Notably, ARRB2-knockout (*ARRB2*KO) lung cancer cells exhibited marked enhancements of cancer migration, invasion, colony formation, and proliferation in response to TLR3 and TLR4 stimulation. Altogether, our current data suggest that ARRB2 can negatively regulate lung cancer progression by inhibiting TLR3- and TLR4-induced autophagy.

## Introduction

Lung cancer is the leading cause of global cancer deaths, accounting for an estimated 2 million diagnoses and 1.8 million deaths [1]. Chronic or persistent inflammation has been considered a high-risk factor for lung cancer disease by promoting cancer development and progression [2, 3]. Recently, it has been demonstrated that toll-like receptors (TLRs) can cause tumor development and progression by orchestrating cellular signaling pathways such as NF-κB signaling, Src/MAPK signaling, Wnt signaling, and phosphoinositide-3 kinase (PI3K)/Akt signaling [4–8]. Consequently, the identification of cellular regulators capable of inhibiting TLR signals might provide novel insight into the development of therapeutic targets for the treatment of lung cancer.

TLRs are highly conserved in evolution and widely expressed on immune cells, where they play an important role in the innate immune system by evoking inflammatory responses [9]. TLRs have been identified on tumor cells, where their activation might orchestrate downstream signaling pathways that serve crucial functions in tumorigenesis and tumor progression [4–8]. Previous studies have demonstrated that expression levels of TLR4, 5, 7, 8, and 9 in non-small-cell lung carcinoma (NSCLC) are markedly higher than those in normal lung tissues [10–13]. Recently, it has been reported that autophagy can facilitate TLR4- and TLR3-triggered migration and invasion of lung cancer cells through the promotion of TRAF6 ubiquitination [13]. These results suggest that the crosstalk between TLR signals and autophagy might play a pivotal role in lung cancer progression.

β-arrestins (βarrs) are multifunctional cellular adaptor proteins that can functionally interact with a large number of cellular proteins including G protein-coupled receptors (GPCRs), thereby playing multiple roles in GPCR signaling, trafficking, and downregulation [14, 15]. There are two types of β-arrestins, β-arrestin 1 (53 kDa) and β-arrestin 2 (46 kDa) located on chromosomes 7 and 11, respectively [16, 17]. β-arrestin 1 (ARRB1) and β-arrestin 2 (ARRB2) have been named for their capacity to sterically hinder GPCRs, ultimately resulting in receptor desensitization [18]. Recently, β-arrestins have been functionally implicated in cancer invasion and metastasis through Src/MAPK signaling, Wnt signaling, NF-κB signaling, and phosphoinositide-3 kinase (PI3K)/Akt signaling pathways [19–23]. β-arrestin 2 (ARRB2) can inhibit hepatocellular carcinoma (HCC) cell migration and invasion through Akt pathway down-regulation [21]. Overexpression of β-arrestins can inhibit the proliferation and motility of triple-negative breast cancer cells by regulating the cell cycle [22]. Regarding lung cancer, it has been reported that β-arrestin 2 can modulate tumorigenesis by regulating inflammation and angiogenesis through activation of CXCR2 and NF-κB in a murine model of lung cancer [23]. Nevertheless, the molecular and cellular mechanisms by which ARRB2 participates in lung cancer progression are poorly understood.

The aim of this study was to determine the functional role of ARRB2 in lung cancer progression through the cross-talk between autophagy and TLR-mediated signaling. To obtain insights into the effect of ARRB2 on lung cancer, microarray data from lung tumor tissues (LTTs) and matched lung normal tissues (mLNTs) of primary non-small cell lung cancer (NSCLC) patients (*n* = 37) were utilized. Associations of ARRB2 expression in LTTs versus matched LNTs were characterized and Gene Set Enrichment Analysis (GSEA) was performed to determine whether ARRB2 expression was enriched in gene sets related to cancers, TLR signaling, and autophagy. Based on the clinical association of ARRB2 and its GSEA in NSCLCs, we investigated the molecular mechanism by which ARRB2 regulated lung cancer progression induced by TLR stimulation and verified the molecular mechanism in ARRB2-knockout (*ARRB2*KO) lung cancer cells generated by CRISPR/Cas9 gene-editing. Taken together, our data demonstrate that ARRB2 down-regulation in lung tumor tissues of NSCLC patients is co-related to gene sets associated with TLR and autophagy signaling pathways. Our results also demonstrate that ARRB2 can negatively regulate the TRAF6-TAB2 signaling axis for NF-κB activation and the TRAF6-BECN1 signaling axis for autophagy induction, thereby attenuating lung cancer progression induced by TLR3 and TLR4.

## Material and methods

### NSCLC patients, tumors, and matched normal specimens

This study was conducted in accordance with the ethical principles stated in the Declaration of Helsinki and approved by the Institutional Review Board (IRB#: 2010-07-204) of Samsung Medical Center (SMC, Seoul, Korea). Written informed consent to use pathological specimens for research was obtained from all patients prior to surgery. Lung tumor tissues (LTTs) and matched lung normal tissues (mLNTs) of NSCLC patients (*n* = 37) were obtained from SMC during surgery. Lung tumor and matched normal specimens of enrolled patients were immediately frozen in liquid nitrogen and stored at −80 °C until use. LTTs and mLNTs were verified by pathologists (Department of Pathology, SMC, Seoul, Korea).

### Cells

Human embryonic kidney (HEK) 293T cells (ATCC, CRL-11268) and 293/hTLR4A (293-htlr-4a, InvivoGen, San Diego, CA, USA) were cultured and maintained in Dulbecco’s modified Eagle’s medium (DMEM; Welgene, LM001-05) supplemented with 10% fetal bovine serum (FBS, R&D Systems, Minneapolis, MN). A549 cells (human lung cancer cell line; ATCC, CCL-185) and H1299 cells (human non-small cell lung cancer cell line; ATCC, CRL-5803) were maintained in RPMI 1640 medium (Welgene, LM011-01) supplemented with 10% fetal bovine serum (FBS), penicillin (100 μg/mL), and streptomycin (100 μg/mL) in a 5% CO_2_ humidified atmosphere at 37 °C.

### Generation of ARRB2-knockout (*ARRB2*KO) cell line with CRISPR/Cas9

To generate *ARRB2*KO lung cancer cells with CRISPR/Cas9 gene editing method, we used two vector systems, sgRNA and cas9 vectors, as previously described [24]. sgRNA and cas9 vectors were kindly provided by Dr. Daesik Kim (Sungkyunkwan University School of Medicine, Suwon, Korea). Briefly, ARRB2-guide RNA sequences for CRISPR/Cas9 were designed on the CRISPR design website (http://crispr.mit.edu/) provided by the Feng Zhang Lab. Guide RNA sequences for ARRB2 were 5’- TGTACTTGGGCAAGCGGGAC-3’ (forward seq.) and 5’- GTCCCGCTTGCCCAAGTACA -3’ (reverse seq.). Complementary oligonucleotides to guide RNAs (gRNAs) were annealed and cloned into a sgRNA vector. sgRNA vector expressing gRNA of ARRB2 and cas9 vector expressing cas9 were transfected into A549 or H1299 cells using Lipofectamine 2000 (Thermo Fisher Scientific, Waltham, MA, USA) according to the manufacturer’s instructions. After two weeks, colonies were isolated from 96-well plates, and expression levels of ARRB2 were analyzed with western blot.

### Antibodies and reagents

Anti-Flag (F3165) and anti-HA (H6908) antibodies were purchased from Sigma-Aldrich (St. Louis, MO, USA). Anti-Myc (sc40), anti-GAPDH (sc47724), anti-BECN1 (sc48341), and anti-Ub (sc-8017) antibodies were purchased from Santa Cruz Biotechnology (Santa Cruz, CA, USA). Anti-TRAF6 (8028S) and anti-LC3 (2775S) were purchased from Cell Signaling Technology (Danvers, MA, USA). Anti-Mouse IgG H&L (HRP) (ab6728) was purchased from Abcam (Cambridge, MA, USA). Goat anti-Rabbit IgG antibody (HRP) (GTX213110-01) was purchased from GeneTex Inc. (Irvine, CA, USA). Lipopolysaccharide (LPS; L3024), dimethyl sulfoxide (DMSO; 472301), paraformaldehyde (P6148), Triton X-100 (T8787), 3-methyladenine (3-MA; M9281), and Dulbecco’s phosphate-buffered saline (DPBS; D8537) were purchased from Sigma-Aldrich (St. Louis, MO, USA). Polyinosinic-polycytidylic acid (Poly(I:C), tlrl-pic) was purchased from InvivoGen (San Diego, CA, USA). Anti-β-arrestin 2 antibody (PA1-732) and Lipofectamine 2000 (11668019) were purchased from Thermo Fisher Scientific (Waltham, MA, USA).

### Plasmid constructs

Flag-TRAF6 (21624) and Flag-BECN1 (24388) plasmids were purchased from Addgene (Cambridge, MA 02142, USA). HA-tagged Ub plasmids were obtained from Dr. J. H. Shim (University of Massachusetts Medical School, USA). Using the Flag-TRAF6 or Flag-BECN1 plasmid as templates, full-length TRAF6 and BECN1 were cloned into pCMV-3Tag-7 (Agilent technologies, 240202) to generate Myc-TRAF6 and Myc-BECN1, respectively. Beta Arrestin 2/ARRB2 cDNA ORF Clone (HG15078-CF) was purchased from Sino Biological US Inc. (Wayne, PA, USA). ARRB2 full length was cloned into a pCMV-3Tag 6 vector (Agilent technologies, 240200) to generate Flag-ARRB2. Truncated mutants of Flag-TRAF6 and Flag-BECN1 were generated as previously described [25, 26].

### Western blotting (WB), Immunoprecipitation (IP), and ubiquitination assay

WB, IP, and ubiquitination assays were performed as previously described [25–35]. Briefly, HEK-293T or 293/hTLR4A cells were seeded into 6-well plates, transfected, and treated as described in the text and Figures. These cells were then incubated for 24 h. After collecting cells, cell lysates were prepared and immunoprecipitated with an anti-Myc or anti-Flag antibody. IP complexes were separated by sodium dodecyl sulfate-polyacrylamide gel electrophoresis (SDS-PAGE, 8–12%) and immune-probed with different antibodies as indicated in the text and Figures. For semi-endogenous IP assays, A549 or H1299 cells were transiently transfected with Flag-ARRB2 and treated with or without LPS (15 μg/mL) for 3 h. IP assay was performed with anti-IgG or anti-Flag antibodies and then immune-probed with anti-Flag, anti-TRAF6, and anti-BECN1 antibodies. For endogenous ubiquitination assays, Ctrl H1299 and *ARRB2*KO H1299 cells were treated with or without LPS (15 μg/mL) for 3 h. IP assay was performed with an anti-TRAF6 or anti-BECN1 antibody and then immune-probed with an anti-TRAF6, anti-BECN1, or anti-Ub antibody. Mock vector, Flag-TRAF6, Myc-TRAF6, Flag-TAB2, Myc-BECN1, HA-Ub, Myc-ARRB2 or Flag-ARRB2 at different concentrations was transfected into HEK293T cells as described in the text and Figures for ubiquitination assays of TRAF6, TAB2, or BECN1. Cell lysates were immunoprecipitated with an anti-Myc or anti-Flag antibody and probed with different antibodies as indicated in the text and Figures. For competitive inhibition assays, mock vector, Myc-BECN1, Flag-TRAF6, and different concentrations of Flag-ARRB2 were transfected into HEK293T cells. Cell lysates were immunoprecipitated with the anti-Myc antibody and probed with anti-Myc and anti-Flag antibodies. Ctrl H1299 and *ARRB2*KO H1299 cells were treated with vehicle (DMSO, 0.1% v/v concentration), PoIy I:C (25 μg/ml), or LPS (15 μg/ml) for 6 h. Cell lysates were immunoblotted with an anti-LC3A/B antibody and an anti-GAPDH (loading control).

### NF-κB luciferase reporter assay

Luciferase reporter assay was performed as previously described [32]. Briefly, 293/hTLR4A cells were transfected with mock vector or different concentrations of Flag-ARRB2, together with the pBIIx-luc NF-κB-dependent reporter construct and the Renilla luciferase vector (Promega, Madison, WI, USA). At 24 h post-transfection, cells were treated or not with LPS (15 μg/ml) for 6 h and lysed. Luciferase activity was measured using a dual luciferase assay kit (Promega).

### Wound-healing migration assay

A wound-healing migration assay was performed following previous protocols [25, 27, 28, 30]. Briefly, Ctrl H1299 and *ARRB2*KO H1299 cells were seeded into 12-well plates and cultured to reach about 90% confluence. Cell monolayers were gently scratched with a sterile pipette tip and washed with a culture medium. After removing floating cells and debris, cells were treated with vehicle (DMSO, 0.1% v/v concentration), Poly I:C (20 μg/ml), or LPS (15 μg/ml) in the presence or absence of 3-MA (5 mM). Cell images were taken at different time points.

### Transwell invasion assay

The transwell invasion assay was performed following previous protocols [25, 27, 28, 30]. Briefly, Ctrl H1299 and *ARRB2*KO H1299 cells were suspended in a culture medium (200 μL) supplemented without FBS and added to the upper compartment of a 24-well Transwell® chamber containing a polycarbonate filter with 8-μm pores and coated with 60 µL of Matrigel (Sigma Aldrich, E1270; 1:9 dilution). Cells were treated with vehicle (DMSO, 0.1% v/v concentration), Poly I:C (15 μg/ml), or LPS (20 μg/ml) in the presence or absence of 3-MA (5 mM) for 24 h. Invaded cells were stained with 0.5% crystal violet (Sigma-Aldrich).

### LC3 puncta assay

LC3 puncta assay was performed as described in previous reports [13, 25, 28]. Briefly, Ctrl H1299 and *ARRB2*KO H1299 cells were grown on glass coverslips, treated with vehicle (DMSO, 0.1% v/v concentration), Poly I:C (5 μg/ml), or LPS (15 μg/ml) in the presence or absence of 3-MA (5 mM) for 6 h, fixed with 4% paraformaldehyde (Sigma, P-6148), and permeabilized with 0.2% Triton X-100 (Sigma, T9284) for 30 min on ice. Cells were incubated with primary rabbit anti-LC3 antibody (1:500 dilution) for overnight, then incubated with Alexa Fluor 488-conjugated donkey anti-rabbit IgG (Molecular Probes, A21206, 1:1000 dilution) for 1 h at room temperature. Slides were mounted in VECTASHIELD mounting medium (Vector Laboratories, H-1000) and examined under an LSM 710 laser-scanning confocal microscope (Carl Zeiss,Jena, Germany).

### Anchorage-independent soft agar colony formation assay

Anchorage-independent soft agar colony formation assay was performed following previous protocols [30, 36]. Briefly, Ctrl H1299 and *ARRB2*KO H1299 cells (1 ×10^4^ cells per well) mixed with 0.3% Difco Noble Agar (BD Biosciences, CA, USA) in a complete medium were plated on the bottom of a 0.5% agar layer in a 6-well plate with complete medium. Culture medium (1.5 mL) with vehicle (DMSO, 0.1% v/v concentration), Poly I:C (20 μg/ml), or LPS (20 μg/ml) in the presence or absence of 3-MA (5 mM) was added to the top of the layer and cells were incubated at 37 °C for four weeks.

### Colony formation assay

The ability of a single cell to grow into a colony was assessed by the colony formation assay as previously described [30, 37, 38]. Briefly, Ctrl H1299 and *ARRB2*KO H1299 cells were harvested with trypsin-EDTA and resuspended as single cells. Cells (1 ×10^3^ cells per well) were plated in 6-well plates and treated with vehicle (DMSO, 0.1% v/v concentration), Poly I:C (25 μg/ml), or LPS (25 μg/ml) in the presence or absence of 3-MA (5 mM). After incubation for 10 days, colonies were stained with 0.5% crystal violet (Sigma-Aldrich) for 30 min at room temperature and the number of colonies was counted.

### MTT assay

To measure cell proliferation, MTT assay was performed as previously described [27]. Briefly, Ctrl H1299 and *ARRB2*KO H1299 cells were seeded in 96-well plates at a density of 1 ×10^3^ cells per well, treated with a vehicle, and incubated in a cell culture incubator overnight. Each well was refreshed with culture media containing vehicle (DMSO, 0.1% v/v concentration), Poly I:C (1 μg/ml), or LPS (7 μg/ml) and incubated for different time periods. After cells were treated with 50 µL of MTT (1 mg/ml) for 4 h, 100 µL of DMSO was added to each well. MTT reduction was quantified by measuring the light absorbance at 595 nm using an ELx800™ absorbance microplate reader (BioTek Instruments, VT, USA). Each test was repeated at least four times in quadruple. Cell viability was calculated using the following equation: Percentage cell viability = [Absorbency (control group) − Absorbency (experiment group)]/Absorbency (control group) ×100%.

### Microarray analysis

Microarray analysis was performed as previously described [26, 39, 40]. Briefly, total RNAs were extracted from tumor tissues and matched normal tissues of 37 patients with NSCLC using Trizol (Thermo Fisher Scientific, 15596026) and purified using RNeasy columns (74106, Qiagen, Hilden, Germany) according to each manufacturer’s protocol. We analyzed mRNA expression using HumanHT-12 expression BeadChips (Illumina, San Diego, CA, USA). Microarray data were pre-processed for background adjustment and normalization with a Bioconductor lumi package (https://bioconductor.org/biocLite.R).

### Gene Set Enrichment Analysis (GSEA)

Different magnitudes (Mags) of ARRB2 expression were obtained from pre-processed microarray data between LTTs (*n* = 37) and matched LNTs (*n* = 37). The top 15 LTTs with downregulated ARRB2 were selected. Genes showing significant differences in expression between 15 LTTs and matched 15 LNTs were analyzed by GSEA (http://www.gsea-msigdb.org/gsea/index.jsp) [41]. Based on different Mags of ARRB2 expression, six LTTs were selected (3 LTTs from the top, Group A and 3 LTTs from the bottom, Group B). GSEA was performed between Group A and Group B to obtain significant differences in gene sets.

### Statistical analysis

All in vitro data are expressed as mean ± standard deviation of triplicate samples or ten different cells. Statistical significance was determined by analysis of variance ANOVA or Student’s *t* test using GraphPad Prism 5.0 (GraphPad Software, San Diego, CA, USA). Values are presented as mean ± SD of three independent experiments. *P*-values were marked as *P < 0.05, **P < 0.01, and ***P < 0.001 in all figures.

## Results

### Experimental design

**Fig. 1:**
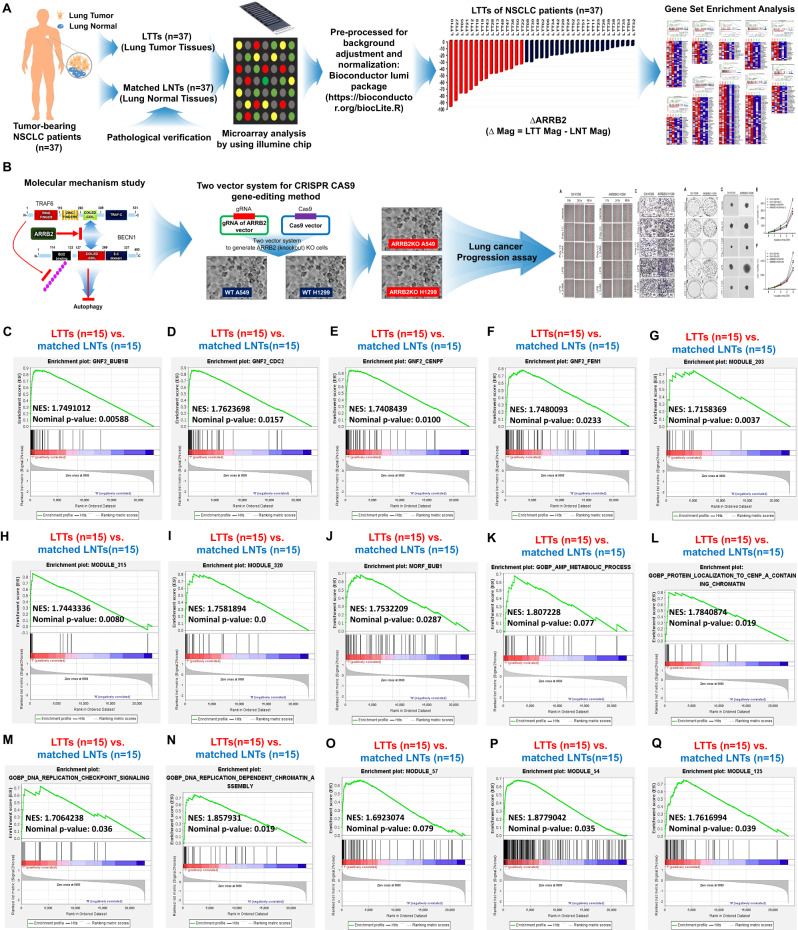
Experimental design and Gene Set Enrichment Analysis (GSEA). **A** Experimental procedures for analyzing microarray data of lung tumor tissues (LTTs) and matched lung normal tissues (mLNTs) of NSCLC patients (*n* = 37). **B** Experimental procedures for verifying the functional role of ARRB2 in lung cancer cells using CRISPR/Cas9 gene-editing method and cancer progression assay. **C**–**Q** According to different magnitudes of ARRB2 in LTTs vs. matched LNTs, top 15 LTTs with down-regulated ARRB2 in 37 LTTs were selected and GSEA (http://www.gsea-msigdb.org/gsea/index.jsp) was performed for 15 LTTs vs. matched 15 LNTs. Fifteen gene sets related to curated, cancer, ontology, or oncogenic gene are presented. NES and nominal p-value are indicated in each inner panel.

Clinical and experimental procedures used in this study are depicted in Fig. 1A, B. To obtain insight into the role of ARRB2 in lung cancer, we obtained lung tumor tissues (LTTs) and matched lung normal tissues (mLNTs) from non-small cell lung cancer (NSCLC) patients (*n* = 37) (Fig. 1A). Microarray analysis was performed with LTTs (*n* = 37) and matched LNTs (*n* = 37) using illumine chip. Data were pre-processed for background adjustment and normalization with a Bioconductor Lumi Package (https://bioconductor.org/biocLite.R). Using pre-processed data, the magnitude difference of ARRB2 (∆ARRB2) was calculated between LTTs and matched LNTs with the following formula: ∆Mag = LTT Mag − mLNT Mag (Supplementary Table S1). For Gene Set Enrichment Analysis (GSEA), we used datasets of LTTs as recommended (https://www.gsea-msigdb.org/gsea/doc/GSEAUserGuideFrame.html). After elucidating the molecular mechanism of β-arrestin 2 (ARRB2), we generated *ARRB2*-knockout (*ARRB2*KO) lung cancer cells using CRISPR/Cas9 gene-editing method (two vector-based systems, Fig. 1B) [24]. Using *ARRB2*KO lung cancer cells, cancer progression assay was performed to verify the cellular function of ARRB2 in lung cancer as depicted in Fig. 1B.

### GSEA between 15 LTTs and matched 15 LNTs

Gene expression profiling interactive analysis data (GEPIA, http://gepia.cancer-pku.cn/detail.php?gene=ARRB2) showed that ARRB2 expression was decreased in lung tumor (Supplementary Fig. S1A, tumor vs. normal) and significantly down-regulated in both lung adenocarcinoma (LUAD) and lung squamous cell carcinoma (LUSC) (Supplementary Fig. S1B, *p < 0.05). Overall survival of LUAD patients was worse for those with low ARRB2 expression (Supplementary Fig. S2, low ARRB2 vs. high ARRB2, *p* = 0.0094), indicating a negative association of ARRB2 expression in lung cancer. To verify these results, we utilized microarray data of NSCLC patients (*n* = 37) and compared ARRB2 expression between LTTs and matched LNTs. The magnitude difference of ARRB2 (∆ARRB2) was decreased in 37 LTTs of NSCLCs (Supplementary Table S1 and Supplementary Fig. S3). To determine whether ARRB2 expression was associated with common pathways and gene sets of cancer, we selected top 15 LTTs with down-regulated ARRB2 (Supplementary Fig. S3, red bars) and performed GSEA (https://www.gsea-msigdb.org) between 15 LTTs and matched 15 LNTs. Fifteen gene sets related to curated cancer, ontology, or oncogenic genes were significantly enriched in 15 LTTs vs. 15 mLNTs (Fig. 1C–Q). Four gene sets, MEIOSIS I CELL CYCLE PROCESS, HOMOLOGOUS CHROMOSOME SEGREGATION, DNA PACKAGING COMPLEX, and REGULATION OF MITOTIC SISTER CHROMATID SEGREGATION, were also enriched in 15 LTTs (Supplementary Fig. S4A–D). These results suggest that ARRB2 downregulation in NSCLCs is associated with cancer phenotype of genes and pathways.

### ARRB2-downregulated LTTs are positively associated with gene sets related to TLR-mediated signaling pathway and autophagy pathway

**Fig. 2:**
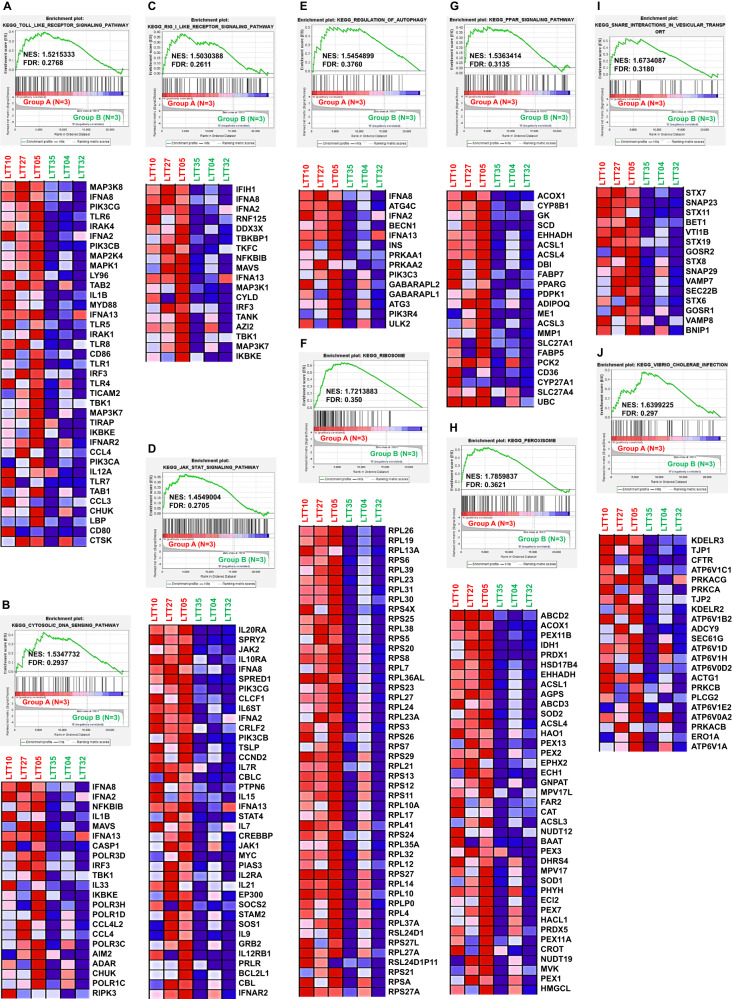
ARRB2-downregulated LTTs are enriched in gene sets related to TLR-mediated signaling pathway and autophagy pathway. **A**–**J** Based on different magnitude of ARRB2 expression in 37 LTTs (Supplementary Fig. S5), 6 LTTs were selected (top 3 LTTs with highly down-regulated ARRB2, Group A and bottom 3 LTTs with lowly down-regulated ARRB2, Group B). GSEA was performed between Group A and Group B to obtain significant differences of gene sets (**A** TOLL LIKE RECEPTOR SIGNALING PATHWAY; **B** CYTOSOLIC DNA SENSING PATHWAY; **C** RIG I LIKE RECEPTOR SIGNALING PATHWAY; **D** JAK STAT SIGNALING PATHWAY; **E** REGULATION OF AUTOPHAGY; **F** RIBOSOME; **G** PPAR SIGNALING PATHWAY; **H** PEROXISOM; **I**, SNARE INTERACTIONS IN VESICULAR TRANSPORT; **J** VIBRIO CHOLERAE INFECTION). Heat map of marker genes for each phenotype is presented in each down. Expression values are presented as colors ranging from red (high expression), pink (moderate), and light blue (low) to dark blue (lowest expression). NES and FDR values were indicated in each inner panel.

It has been reported that the association of ARRB2 and TRAF6 can negatively regulate toll-like receptor-interleukin 1 receptor signaling [42]. In addition, a previous report has shown that autophagy can facilitate TLR4- and TLR3-triggered migration and invasion of lung cancer cells by promoting TRAF6 ubiquitination [13]. Based on these results, we determined whether ARRB2 could negatively regulate autophagy induced by TLR signals through the regulation of TRAF6 ubiquitination, thereby inhibiting the migration and invasion of lung cancer. To preliminarily examine this possibility, six LTTs were selected from 37 LTTs according to ARRB2 expression (Supplementary Fig. S5: Group A, 3 LTTs with highly down-regulated ARRB2; Group B, 3 LTTs with lowly down-regulated ARRB2). GSEA was performed between Group A and Group B. Notably, three innate receptor-related gene sets, TOLL LIKE RECEPTOR SIGNALING PATHWAY, CYTOSOLIC DNA SENSING PATHWAY, and RIG I LIKE RECEPTOR SIGNALING PATHWAY, were significantly enriched in Group A LTTs vs. Group B LTTs (Fig. 2A, TOLL LIKE RECEPTOR SIGNALING PATHWAY; Fig. 2B, CYTOSOLIC DNA SENSING PATHWAY; Fig. 2C, RIG I LIKE RECEPTOR SIGNALING PATHWAY). A gene set of JAK STAT SIGNALING PATHWAY was also enriched in Group A (Fig. 2D). Furthermore, six gene sets related to autophagocytosis (i.e., REGULATION OF AUTOPHAGY, PPAR SIGNALING PATHWAY, RIBOSOME, PEROXISOM, SNARE INTERACTIONS IN VESICULAR TRANSPORT, and VIBRIO CHOLERAE INFECTION) were significantly enriched in Group A LTTs vs. Group B LTTs (Fig. 2E, REGULATION OF AUTOPHAGY; Fig. 2F, RIBOSOME; Fig. 2G, PPAR SIGNALING PATHWAY; Fig. 2H, PEROXISOM; Fig. 2I, SNARE INTERACTIONS IN VESICULAR TRANSPORT; Fig. 2J, VIBRIO CHOLERAE INFECTION). These GSEA results suggest that ARRB2 downregulation might be positively associated with gene sets related to TLR-mediated signaling and autophagy pathway.

### ARRB2 interacts with TRAF6 and BECN1

**Fig. 3:**
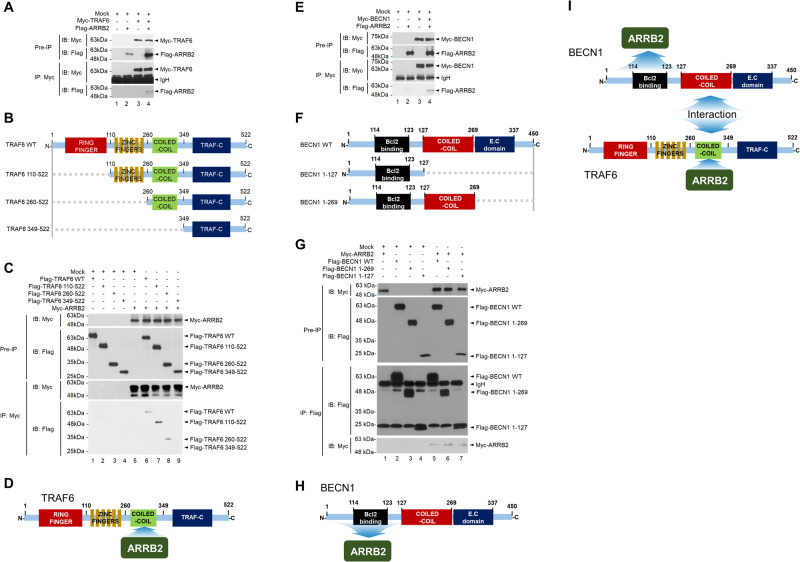
ARRB2 interacts with TRAF6 and BECN1. **A** HEK-293T cells were transfected with mock, Myc-TRAF6, and Flag-ARRB2. Immunoprecipitation (IP) assay was performed with an anti-Myc antibody. IP complexes were immune-probed with anti-Myc and anti-Flag antibodies. **B** Truncated mutants of TRAF6 were generated as described in Materials and Methods. **C** HEK-293T cells were transfected with mock, Myc-ARRB2, Flag-TRAF6 wild type (WT), and Flag-truncated mutants of TRAF6 as indicated. IP assay was performed with an anti-Myc antibody. IP complexes were immune-probed with anti-Myc and anti-Flag antibodies. **D** A schematic model of the interaction between TRAF6 and ARRB2. **E** HEK-293T cells were transfected with mock, Myc-BECN1, and Flag-ARRB2 and IP assay was performed with an anti-Myc antibody. IP complexes were immune-probed with anti-Myc and anti-Flag antibodies. **F** Truncated mutants of BECN1 were generated as described in Material and Methods. **G** HEK-293T cells were transfected with mock, Myc-ARRB2, Flag-BECN1 wild type (WT), and Flag-truncated mutants of BECN1 as indicated. IP assay was performed with an anti-Flag antibody. IP complexes were immune-probed with anti-Myc and anti-Flag antibodies. **H** A schematic model of the interaction between BECN1 and ARRB2. **I** A molecular model of the interaction between ARRB2 and TRAF6 or BECN1.

Based on GSEA results, we explored the molecular mechanism by which ARRB2 was implicated in TLR-mediated signaling and autophagy pathway. The ubiquitination of TRAF6 and BECN1 plays a key role for the induction of autophagy in response to TLR3 and TLR4 stimulation [13, 26–28]. Thus, we examined whether ARRB2 affected the association of TRAF6 with BECN1, thereby affecting the ubiquitination of TRAF6 and BECN1. Myc-TRAF6 specifically interacted with Flag-ARRB2 (Fig. 3A, lane 4). To determine the interaction site of TRAF6 with ARRB2, truncated mutants of TRAF6 were generated (Fig. 3B) and an immunoprecipitation (IP) assay was performed with ARRB2. Myc-ARRB2 interacted with Flag-TRAF6 wild type (WT), Flag-TRAF6 110-522, and Flag-TRAF6 260-522 (Fig. 3C, lane 6-8), but not with Flag-TRAF6 349-522 (Fig. 3C, lane 9), indicating that ARRB2 interacted with the coiled-coil domain of TRAF6 (Fig. 3D). Flag-ARRB2 interacted with Myc-BECN1 (Fig. 3E, lane 4). IP assay with truncated mutants of BECN1 (Fig. 3F) revealed that Flag-BECN1 WT, Flag-BECN1 1-269, and Flag-BECN1 1-127 interacted with Myc-ARRB2 (Fig. 3G, lane 5-7), indicating that ARRB2 could interact with the N-terminus (BECN1 1-127) of BECN1 (Fig. 3H). These results indicate that ARRB2 can interact with TRAF6 and BECN1 as depicted in Fig. 3I.

### ARRB2 inhibits ubiquitination of TRAF6 and BECN1

**Fig. 4:**
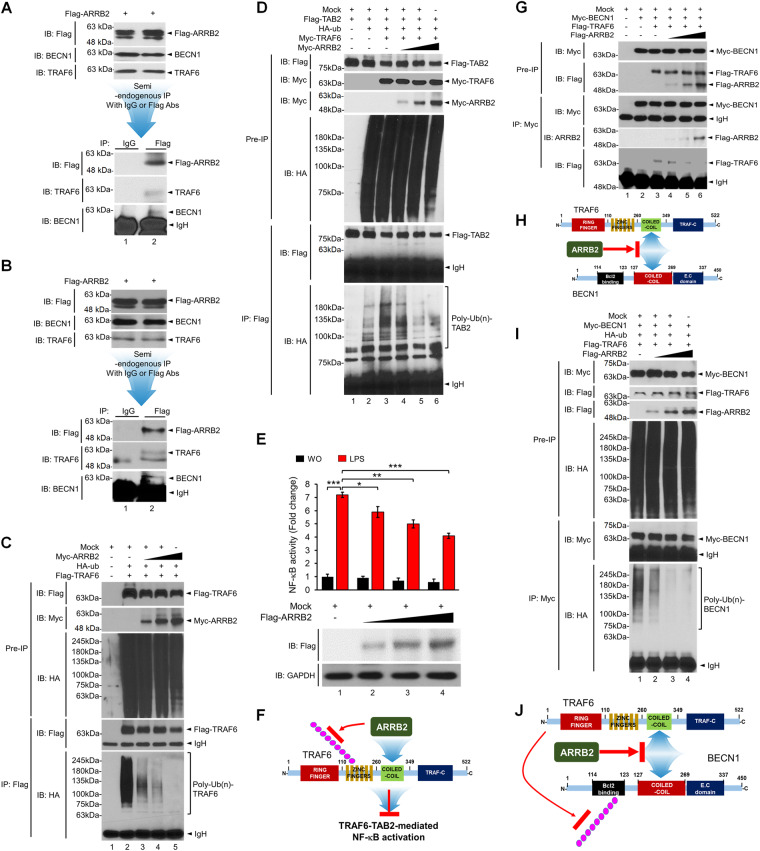
ARRB2 inhibits ubiquitination of TRAF6 and BECN1. A549 (**A**) and H1299 (**B**) lung cancer cells were transiently transfected with Flag-ARRB2. Semi-endogenous immunoprecipitation (IP) assay was performed with anti-IgG and anti-Flag antibodies. IP complexes were immune-probed with anti-Flag, anti-TRAF6, and anti-BECN1 antibodies. **C** HEK-293T cells were transfected with mock, HA-Ub, Flag-TRAF6, and different concentrations of Myc-ARRB2 as indicated. IP assay was performed with anti-Flag antibody. IP complexes were immune-probed with anti-Myc, anti-Flag, and anti-HA antibodies. **D** HEK-293T cells were transfected with mock, Flag-TAB2, HA-Ub, Myc-TRAF6, and different concentrations of Myc-ARRB2 as indicated. IP assay was performed with an anti-Flag antibody. IP complexes were immune-probed with anti-Myc, anti-Flag, and anti-HA antibodies. **E** 293/hTLR4A cells were transfected with mock vector or different concentrations of Flag-ARRB2 together with a pBIIx-luc NF-κB-dependent reporter construct and a Renilla luciferase vector and then treated with or without LPS (15 μg/ml) for 6 h. Luciferase activity was measured as described in Material and Methods. Flag-ARRB2 expression was verified by anti-Flag antibody, along with anti-GAPDH as a loading control. Results are presented as mean ± SD of three independent experiments. *p < 0.05, **p < 0.01, and ***p < 0.001. **F** A schematic model of the inhibition of ARRB2 in TRAF6-TAB2-mediated NF-κB activation. **G** HEK-293T cells were transfected with mock, Myc-BECN1, Flag-TRAF6, and different concentrations of Flag-ARRB2, as indicated. IP assay was performed with an anti-Myc antibody. IP complexes were immune-probed with anti-Myc, anti-Flag, and anti-ARRB2 antibodies. **H** A schematic model of the inhibition of ARRB2 in the interaction between TRAF6 and BECN1. **I** HEK-293T cells were transfected with mock, Myc-BECN1, HA-Ub, Flag-TRAF6, and different concentrations of Flag-ARRB2 as indicated. IP assay was performed with an anti-Myc antibody. IP complexes were immune-probed with anti-Myc, anti-HA, and anti-Flag antibodies. **J** A schematic model of how ARRB2 interrupts the association between TRAF6 and BECN1, thereby inhibiting BECN1 ubiquitination.

To verify the molecular association of the ARRB2-TRAF6-BECN1 complex, we performed a semi-endogenous IP assay in A549 and H1299 human lung cancer cells. A549 or H1299 cells were transiently transfected with the Flag-ARRB2 vector. IP assay was then performed with an anti-Flag antibody. Flag-ARRB2 interacted with endogenous TRAF6 and BECN1 in both cells as compared to those of control IgG (Fig. 4A, A549; Fig. 4B, H1299), indicating that ARRB2 was endogenously associated with TRAF6 and BECN1. The coiled-coil domain of TRAF6 is essential for its auto-ubiquitination through the dimerization of TRAF6 [43]. It can induce TAB2 ubiquitination for NF-κB activation and simultaneously induce autophagy through BECN1 ubiquitination [13, 43–45]. Since ARRB2 interacted with the coiled-coil domain of TRAF6 (Fig. 3C, D), we examined whether ARRB2 affected the ubiquitination of TRAF6 and TAB2 for NF-κB activation induced by TLR4 stimulation. Results showed that ubiquitination of TRAF6 was induced in the absence of ARRB2, whereas markedly inhibited in the presence of ARRB2 (Fig. 4C, lane 2 vs. lanes 3-5). Importantly, TAB2 ubiquitination by TRAF6 was significantly enhanced in the absence of ARRB2 (Fig. 4D, lane 3), whereas markedly inhibited in the presence of ARRB2 (Fig. 4D, lane 3 vs. lanes 4-6). Moreover, NF-κB activation induced by TLR4 was significantly attenuated in the presence of ARRB2 (Fig. 4E, lane 1 vs. lanes 2-4), indicating that the interaction of ARRB2 with the coiled-coil domain of TRAF6 could inhibit the ubiquitination of TRAF6 and TAB2, thereby attenuating NF-κB activation as depicted in Fig. 4F.

Next, we examined whether ARRB2 affected the ubiquitination of BECN1. We have previously reported that TRAF6 can interact with the coiled-coil domain of BECN1 and induce BECN1 ubiquitination [25, 34]. We found that ARRB2 interacted with the coiled-coil domain of TRAF6 (Fig. 3C, D). Thus, we examined whether ARRB2 affected the interaction between BECN1 and TRAF6. BECN1 interacted with TRAF6 in the absence of ARRB2 (Fig. 4G, lane 3), whereas significant attenuations of BECN1-TRAF6 interaction could be observed in the presence of ARRB2 in a dose-dependent manner (Fig. 4G, lane 3 vs. lanes 4-6). Additionally, the interaction between BECN1 and ARRB2 gradually increased (Fig. 4G, lanes 4-6 in IB with ARRB2). These results suggest that ARRB2 could interrupt the molecular association of TRAF6 with BECN1 (Fig. 4H). Notably, BECN1 ubiquitination was significantly induced in the absence of ARRB2 (Fig. 4I, lane 1), whereas marked inhibitions could be observed in the presence of ARRB2 in a dose-dependent manner (Fig. 4I, lane 1 vs. lanes 2-4). These results suggest that ARRB2 can interrupt the interaction between TRAF6 and BECN1 and inhibit BECN1 ubiquitination as depicted in Fig. 4J.

### ARRB2 attenuates autophagy induced by TLR3 and TLR4 stimulation

**Fig. 5:**
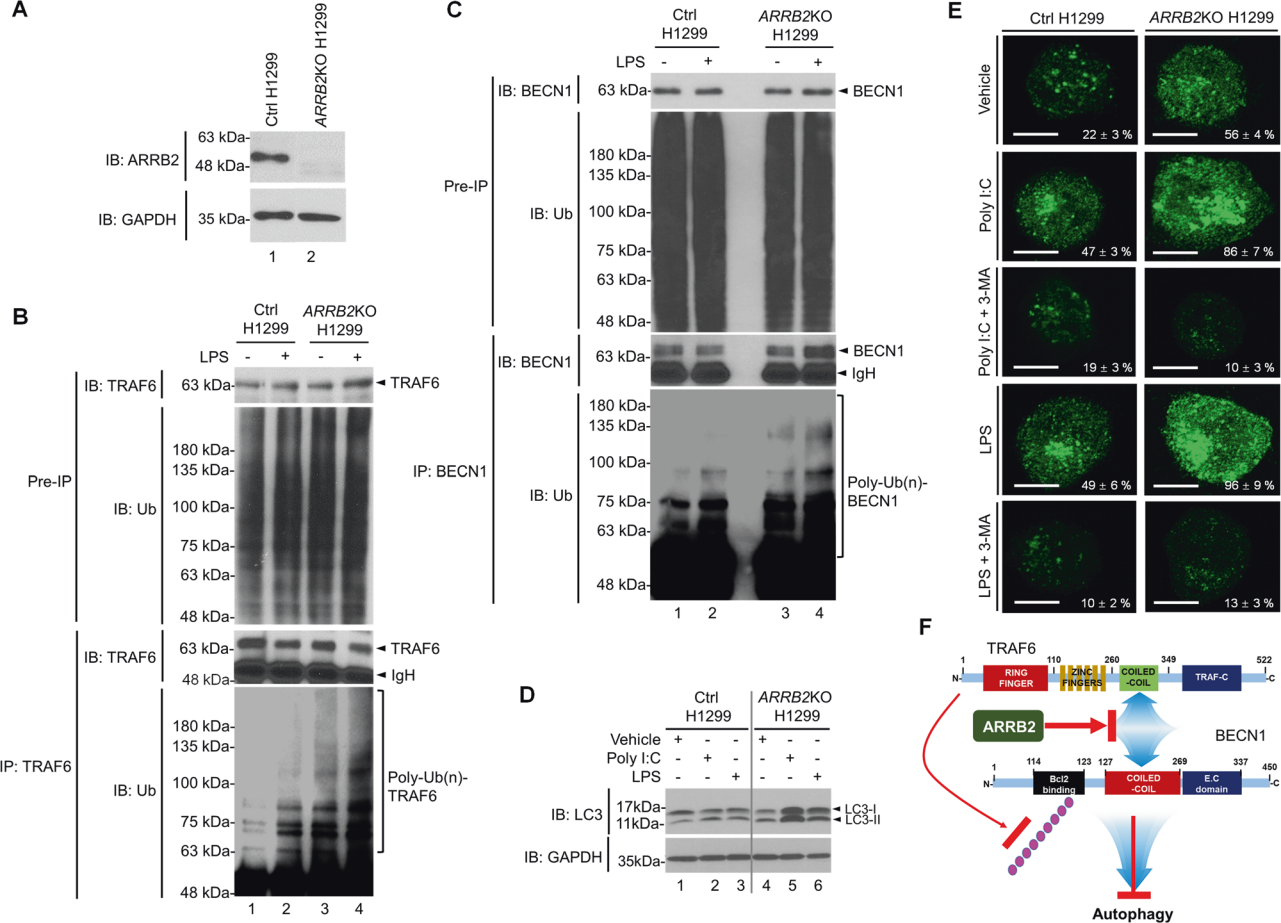
ARRB2 negatively regulates autophagy induced by TLR3 and TLR4. **A** ARRB2-knockout (*ARRB2*KO) H1299 cells were generated by CRISPR/Cas9 gene-editing method as described in Material and Methods. ARRB2 expression was verified by anti-ARRB2 antibody, along with anti-GAPDH as a loading control. **B**, **C** Control (Ctrl) H1299 and *ARRB2*KO H1299 cells were treated with or without LPS (15 μg/ml) for 3 h. Endogenous immunoprecipitation (IP) assay was performed with anti-TRAF6 (**B**) or anti-BECN1 (**C**). IP complexes were immune-probed with anti-TRAF6, anti-BECN1, or anti-Ub antibody. **D** Ctrl H1299 and *ARRB2*KO H1299 cells were treated with vehicle (DMSO, 0.1% v/v concentration), PoIy I:C (25 μg/ml), LPS (15 μg/ml) for 6 h. Cell lysates were immunoblotted with an anti-LC3A/B antibody and anti-GAPDH (as a loading control). **E** Ctrl H1299 and *ARRB2*KO H1299 cells were treated with vehicle (DMSO, 0.1% v/v concentration), Poly I:C (5 μg/ml), or LPS (15 μg/ml) in the presence or absence of 3-MA (5 mM) for 6 h, and immunolabeled with LC3 antibody, as described in Material and Methods. Images shown are representative fluorescence confocal microscopic photographs. Quantification of the percentage of cells with autophagosomes is shown (±SD, *n* = 50 cells). Scale bar: 10 μm. **F** A schematic model of how ARRB2 interrupts the association between TRAF6 and BECN1 and inhibits autophagy through inhibition of BECN1 ubiquitination.

**Fig. 6:**
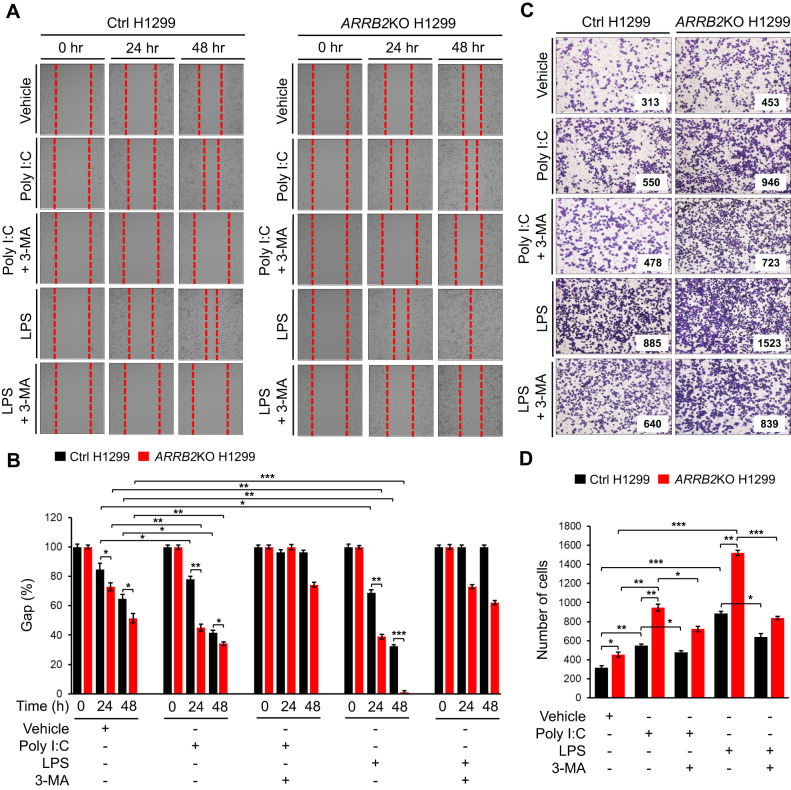
*ARRB2*KO H1299 cells exhibit enhanced cell migration and invasion induced by TLR3 and TLR4. **A**, **B** Ctrl H1299 and *ARRB2*KO H1299 cells were treated with vehicle (DMSO, 0.1% v/v concentration), Poly I:C (20 μg/ml), or LPS (15 μg/ml) in the presence or absence of 3-MA (5 mM) for different time periods (**A**), as described in Materials and Methods. The residual gap between migrating cells from the opposing wound edge is expressed as a percentage of the initial scraped area (±SD, *n* = 3 different plates) (**B**). *p < 0.05, **p < 0.01, and ***p < 0.001. **C**, **D** Ctrl H1299 and *ARRB2*KO H1299 cells were treated with vehicle (DMSO, 0.1% v/v concentration), Poly I:C (15 μg/ml), or LPS (20 μg/ml) in the presence or absence of 3-MA (5 mM) (**C**), as described in Material and Methods. The number of migrating cells were counted. Results are presented as the mean ± SD of three independent experiments (**D**). *p < 0.05, **p < 0.01, and ***p < 0.001.

BECN1 ubiquitination plays a pivotal role in autophagy induction by TLR3 and TLR4 stimulation [13, 43–45]. Given the above results that ARRB2 inhibited the ubiquitination of TRAF6 and BECN1 (Fig. 4), we examined whether ARRB2 negatively regulated TLR3- and TLR4-induced autophagy in lung cancer cells. We generated ARRB2-knockout (*ARRB2*KO) H1299 and A549 lung cancer cells using CRISPR/Cas9 gene-editing method (Fig. 5A, *ARRB2*KO H1299; Supplementary Fig. S6A, *ARRB2*KO A549). To see whether deficiency of ARRB2 enhanced the ubiquitination of TRAF6 and BECN1 for autophagy induction, *ARRB2*KO H1299 cells were treated with or without LPS. IP assay was then performed with an anti-TRAF6 or anti-BECN1 antibody. The ubiquitination of TRAF6 or BECN1 was significantly induced in the presence of LPS in Ctrl H1299 cells (Fig. 5B, lane 2, TRAF6; Fig. 5C, lane 2, BECN1). Importantly, the ubiquitination of TRAF6 or BECN1 was markedly elevated in the presence of LPS in *ARRB2*KO H1299 cells as compared to those in Ctrl H1299 cells (Fig. 5B, lane 2 vs. lane 4, TRAF6; Fig. 5C, lane 2 vs. lane 4, BECN1). Moreover, LC3-II levels were induced by TLR3 and TLR4 stimulation in Ctrl H1299 cells (Fig. 5D, lane 2 and 3) and markedly enhanced in *ARRB2*KO H1299 cells (Fig. 5D, lane 5 and 6). To verify these results, LC3 puncta assay was performed using Ctrl H1299 and *ARRB2*KO H1299 cells. As expected, LC3 puncta was increased in the presence of Poly I:C or LPS in both cells, whereas it was markedly inhibited in the presence of 3-MA, an autophagy inhibitor (Fig. 5E). Notably, LC3 puncta was greatly enhanced in *ARRB2*KO H1299 cells treated with Poly I:C or LPS as compared to that in Ctrl H1299 cells (Fig. 5E, *ARRB2*KO H1299 treated with Poly I:C or LPS vs. Ctrl H1299 treated with Poly I:C or LPS). Similar results were observed in *ARRB2*KO A549 cells (Supplementary Fig. S6B). As depicted in Fig. 6F, these results suggest that ARRB2 can interrupt the molecular interaction between TRAF6 and BECN1 and inhibit BECN1 ubiquitination, leading to the attenuation of autophagy.

### ARRB2KO lung cancer cells exhibit enhanced lung cancer progression induced by TLR3 and TLR4

Next, we examined whether ARRB2 was involved in lung cancer progression induced by TLR3 and TLR4. Ctrl H1299 and *ARRB2*KO H1299 cells were treated with vehicle, Poly I:C, and LPS in the presence or absence of an autophagy inhibitor (3-MA) for different time periods. Cell migration assay was then performed. Cancer cell migration was increased in Ctrl H1299 cells in the presence of Poly I:C or LPS, and significantly elevated in *ARRB2*KO H1299 cells (Fig. 6A, B, Ctrl H1299 vs. *ARRB2*KO H1299 treated with Poly I:C or LPS). As expected, significant inhibitions of cancer cell migration were observed after cells were co-treated with Poly I:C plus 3-MA or LPS plus 3-MA (Fig. 6A, B, Ctrl H1299 vs. *ARRB2*KO H1299 treated with Poly I:C plus 3-MA or LPS plus 3-MA). Moreover, the invasion ability of *ARRB2*KO H1299 cancer cells was significantly enhanced as compared to that of Ctrl H1299 cells (Fig. 6C, D, Ctrl H1299 vs. *ARRB2*KO H1299). Consistent results were found for *ARRB2*KO A549 cells vs. Ctrl A549 (Supplementary Fig. S7).

**Fig. 7:**
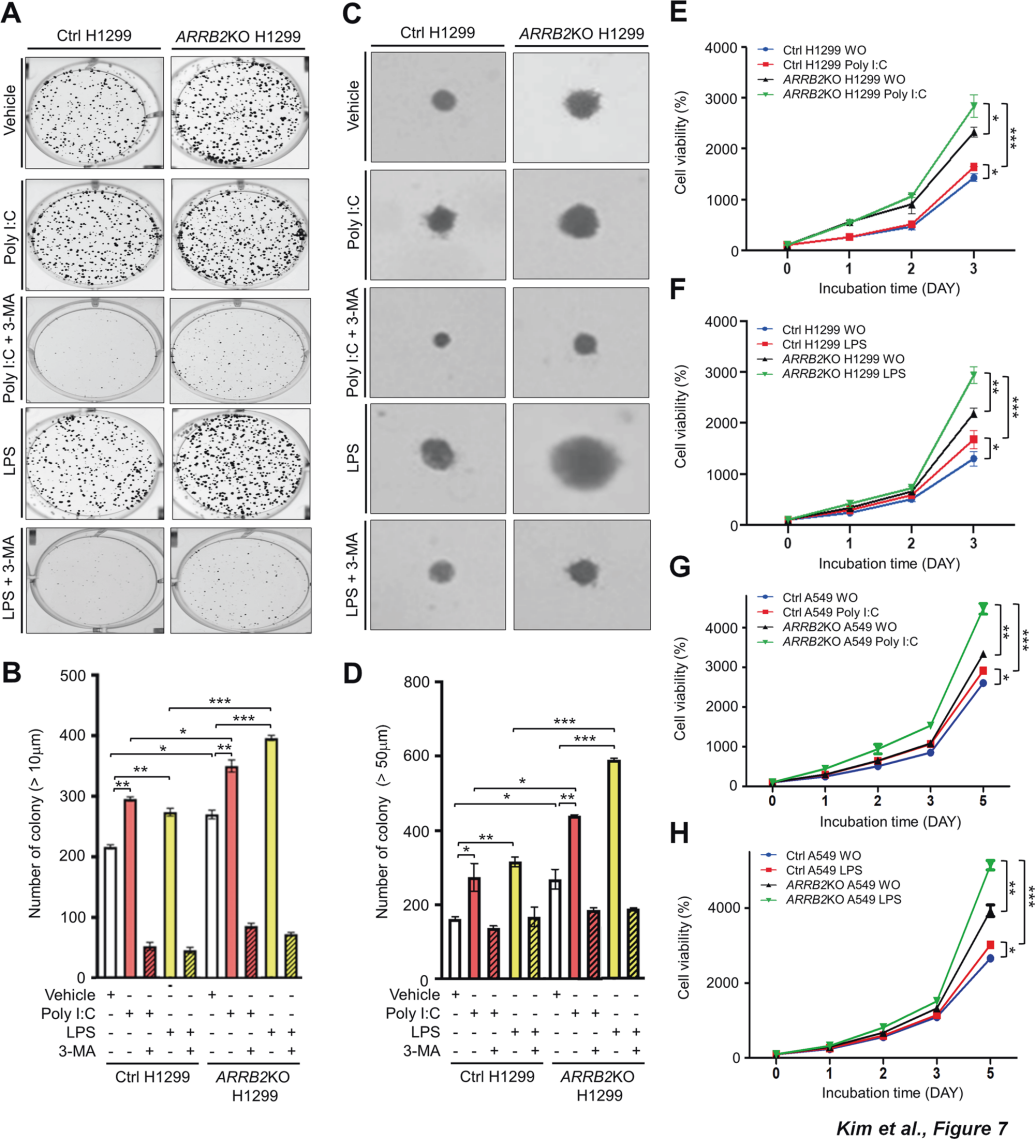
*ARRB2*KO H1299 cells exhibit enhancements of colony formation and proliferation by TLR3 and TLR4. **A**, **B** Ctrl H1299 and *ARRB2*KO H1299 cells were treated with vehicle (DMSO, 0.1% v/v concentration), Poly I:C (25 μg/ml), or LPS (25 μg/ml) in the presence or absence of 3-MA (5 mM). Anchorage-dependent colony formation assay was performed (**A**), as described in Materials and Methods. The number of colonies was measured using Adobe Photoshop software, and results are presented as mean ± SD of three independent experiments (**B**, *n* = 3 plates). *p < 0.05, **p < 0.01, and ***p < 0.001. **C**, **D** Ctrl H1299 and *ARRB2*KO H1299 cells were treated with vehicle (DMSO, 0.1% v/v concentration), Poly I:C (20 μg/ml), or LPS (20 μg/ml) in the presence or absence of 3-MA (5 mM). Anchorage-independent colony formation assay was performed (**C**), as described in Materials and Methods. **D** The number of colonies was measured using Adobe Photoshop software, and results are presented as mean ± SD of three independent experiments (**D**, *n* = 3 plates). *p < 0.05, **p < 0.01, and ***p < 0.001. Ctrl H1299 and *ARRB2*KO H1299 cells (**E**, Poly I:C; **F**, LPS) or Ctrl A549 and *ARRB2*KO A549 cells (**G**, Poly I:C; **H**, LPS) were treated with vehicle (WO, DMSO, 0.1% v/v concentration), Poly I:C (1 μg/ml), or LPS (7 μg/ml). MTT assay was then performed, as described in in Material and Methods. Results are presented as the mean ± SD of three independent experiments. *p < 0.05, **p < 0.01, and ***p < 0.001.

We further evaluated the ability of cancer formation using anchorage-dependent or -independent colony-forming and cell proliferation assays. The number of colonies was increased in both Ctrl H1299 and *ARRB2*KO H1299 cells treated with Poly I:C or LPS as compared to that in cells treated with vehicle (Fig. 7A–D). It was significantly higher in *ARRB2*KO H1299 cells than in Ctrl H1299 cells (Fig. 7A–D, *ARRB2*KO H1299 vs. Ctrl H1299 treated with Poly I:C or LPS). However, co-treatment with 3-MA, an autophagy inhibitor, resulted in a marked inhibition in the number of colonies in both cells (Fig. 7A–D, Ctrl H1299 and *ARRB2*KO H1299 treated with Poly I:C plus 3-MA or LPS plus 3-MA vs. Ctrl H1299 and *ARRB2*KO H1299 treated with Poly I:C or LPS). In addition, the proliferation of *ARRB2*KO H1299 or *ARRB2*KO A549 cancer cells was significantly increased compared to that of Ctrl H1299 or Ctrl A549 cells (Fig. 7E, F, *ARRB2*KO H1299 WO vs. Ctrl H1299 WO; Fig. 7G, H, *ARRB2*KO A549 WO vs. Ctrl A549 WO). The proliferation of *ARRB2*KO H1299 or *ARRB2*KO A549 cells was enhanced after treatment with Poly I:C or LPS compared to that of Ctrl H1299 or Ctrl A549 cells treated with Poly I:C or LPS (Fig. 7E, F, *ARRB2*KO H1299 treated with Poly I:C or LPS vs. Ctrl H1299 treated with Poly I:C or LPS; Fig. 7G, H, *ARRB2*KO A549 treated with Poly I:C or LPS vs. Ctrl A549 treated with Poly I:C or LPS). To further verify the functional roles of ARRB2 in lung cancer progression, H1299 cells were transfected with Flag-ARRB2 or Mock as a control vector (Supplementary Fig. S8A), and then cancer migration, invasion, colony formation, and proliferation assay were performed. The migration, invasion, and colony formation were significantly attenuated in the Flag-ARRB2-overexpressed H1299 cells in response to Poly I:C or LPS stimulation, as compared to those of H1299 cells transfected with the mock as a control vector (Supplementary Fig. S8B, C, Flag-ARRB2 H1299 vs. Mock H1299, migration; Supplementary Fig. S9A, B, Flag-ARRB2 H1299 vs. Mock H1299, invasion; Supplementary Fig. S9C, D, Flag-ARRB2 H1299 vs. Mock H1299, colony formation). Moreover, the ability of cell proliferation was consistently inhibited in ARRB2-overexpressed H1299 cells in response to Poly I:C or LPS stimulation, as compared to those of H1299 cells transfected with the mock as a control vector (Supplementary Fig. S10, Flag-ARRB2 H1299 vs. Mock H1299). Taken together, these results suggest that ARRB2 can functionally inhibit the migration, invasion, colony formation, and proliferation of lung cancer cells induced by TLR3 and TLR4 stimulation.

## Discussion

**Fig. 8:**
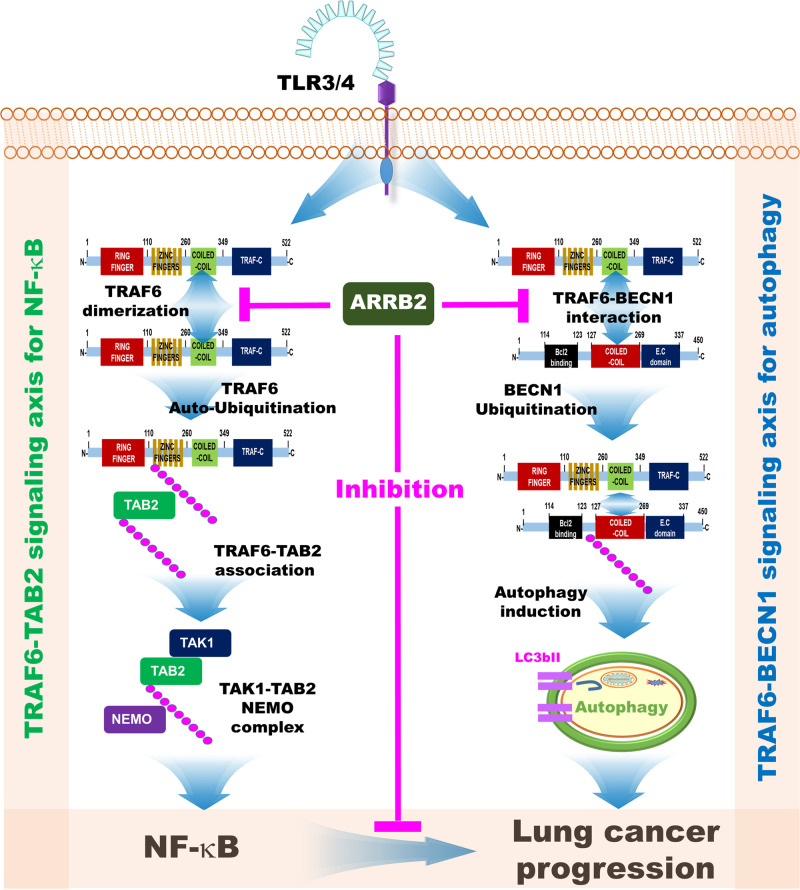
A schematic model showing how ARRB2 regulates lung cancer progression by regulating the TRAF6-TAB2 signaling axis for the activation of NF-κB and the TRAF6-BECN1 signaling axis for the induction of autophagy. Engagement of TLR3/TLR4 can induce the activation of NF-κB through the TRAF6-TAB2 signaling axis (left, green) and autophagy through the TRAF6-BECN1 signaling axis (right, blue), eventually facilitating lung cancer progression. ARRB2 interacts with the coiled-coil domain of TRAF6 and inhibits TRAF6 auto-ubiquitination and TRAF6-TAB2 association for the activation of NF-κB (left, pink line). Simultaneously, ARRB2 interrupts the interaction between TRAF6 and BECN1 and inhibits BECN1 ubiquitination for autophagy induction (right, pink line). Consequently, ARRB2 inhibits lung cancer progression regulated by two signaling axes, TRAF6-TAB2 signaling axis for NF-κB activation and TRAF6-BECN1 signaling axis for autophagy induction, in response to TLR3 and TLR4 stimulation (center, pink line).

In this study, we demonstrated that ARRB2, a multifunctional cellular adaptor protein capable of regulating cellular signaling pathways [14–23], could functionally regulate lung cancer progression induced by TLR3 and TLR4 through inhibition of NF-κB and autophagy. Importantly, the logical background and initiative idea of the functional role of ARRB2 in lung cancer could be found in TCGA data and microarray data of NSCLC patients (*n* = 37). ARRB2 expression was significantly down-regulated in lung cancers. Its downregulation was significantly enriched in gene sets related to cancer, TLRs, and autophagy pathway. Based on these results, we evaluated the possibility that ARRB2 might be functionally implicated in lung cancer progression specifically regulated by TLRs-induced autophagy. We experimentally determined molecular and cellular mechanisms by which ARRB2 could regulate lung cancer progression. As proposed in Fig. 8, our data demonstrated that ARRB2 could functionally inhibit TRAF6-TAB2 signaling and TRAF6-BECN1 signaling for coordinating lung cancer progression through NF-κB activation and autophagy, respectively. Considering recent studies reporting that TLR-mediated signaling can orchestrate downstream signaling pathways to serve crucial functions in tumorigenesis and tumor progression [2–6], our results might provide potential strategies for the development of therapies that target TLR signaling pathways for the treatment of lung cancer.

Although the functional role of TLRs in the tumor microenvironment has been well defined in terms of inducing persistent inflammation [2–5], the roles of TLRs in tumor cells remain unclear. In the case of NSCLC, several TLRs such as TLR4, TLR5, TLR7, TLR8, and TLR9 have been found to be markedly higher in NSCLC tissues than in normal lung tissues [4, 10–12]. TLRs can regulate the growth of lung cancer cells and promote lung cancer progression [4]. It has been shown that TLR4 can induce cancer cell proliferation and tumor growth in vivo by coordinating PI3K/AKT signaling pathway [46]. Engagements of TLR7 or TLR8 agonists in lung cancer cells can induce the activation of NF-κB and upregulation of Bcl-2 expression, leading to increases in cell survival and resistance to apoptosis [47]. Moreover, stimulation with TLR4 and TLR3 can induce the production of IL-6, CCL2/MCP-1, CCL20/MIP-3α, VEGFA (vascular endothelial growth factor A), and MMP2 known to play pivotal roles in the migration and invasion of lung cancer cells through induction of autophagy [13]. Importantly, inhibition of autophagy can induce impairments of TLR3- and TLR4-triggered activation of MAPK and NF-κB signaling pathways through inhibition of TLR3/4-induced TRAF6 ubiquitination and MAP3K7 (TAK1) activation [13]. In line with the report [13], the engagement of TLR4 induced the interaction between BECN1 and TRAF6, and led to the modification of BECN1 by the addition of K63-linked ubiquitin chains by TRAF6 [48]. Importantly, the ubiquitination of BECN1 on K117 as a key site for linking ubiquitin chains induced the multimerization of BECN1 and the increase of PI3KC3 enzymatic activity, thereby leading to the amplification of TLR-induced autophagy [48]. These results strongly suggest that TLR3/4 stimulation can induce the TRAF6-TAK1 signaling axis for NF-κB activation and the TRAF6-BECN1 signaling axis for autophagy induction, thereby facilitating lung cancer progression.

In the current study, we found that ARRB2 was functionally involved in these two signaling axes (i.e., TRAF6-TAB2 and TRAF6-BECN1 signaling axes) for NF-κB activation and autophagy induction in response to TLR3/4 stimulation. First, ARRB2 interacted with the coiled-coil domain of TRAF6 and inhibited TRAF6 ubiquitination, which is critical for the activation of TRAF6-downstream signaling for the activation of NF-κB through ubiquitination of TAB2 and formation of TAB2-TAK1-NEMO complex [44, 49]. Consistently, inhibition of TRAF6 ubiquitination by ARRB2 led to attenuation of TAB2 ubiquitination, resulting in inhibition of NF-κB induced by TLR4. These results suggest that ARRB2 can inhibit the TRAF6-TAB2 signaling axis for the activation of NF-κB as depicted in Fig. 8 (left). Second, the interaction between the coiled-coil domain of TRAF6 and ARRB2 interrupted the molecular association of TRAF6-BECN1 proteins for autophagy induction. Overexpression of ARRB2 competitively inhibited the interaction between TRAF6 and BECN1, thereby markedly attenuating BECN1 ubiquitination. Notably, ubiquitination levels of BECN1 and TRAF6 were remarkably enhanced in *ARRB2*KO lung cancer cells, strongly indicating that ARRB2 could inhibit the TRAF6-BECN1 signaling axis for autophagy induction.

Given the molecular mechanism by which ARRB2 inhibited the TRAF6-related signaling axis for the activation of NF-κB and autophagy induction, we verified the functional role of ARRB2 in lung cancer progression. Consistent with mechanistic results, NSCLCs with down-regulated ARRB2 exhibited significant enrichments of TLRs and autophagy-related gene sets. In terms of function aspects, *ARRB2*KO lung cancer cells showed marked enhancements of autophagy and cancer progression including migration, invasion, colony formation, and proliferation in response to TLR3 and TLR4 stimulation. As illustrated in Fig. 8, based on clinical observations presented in Figs. 1 and 2, this study explored the molecular and cellular mechanisms by which ARRB2 was negatively implicated in lung cancer progression induced by TLR3 and TLR4 and proposed a possible model. Engagements of TLR3/TLR4 can induce the activation of NF-κB through the TRAF6-TAB2 signaling axis (Fig. 8, left) and autophagy through the TRAF6-BECN1 signaling axis (Fig. 8, right), eventually facilitating lung cancer progression [13, 43–45]. ARRB2 can interact with the coiled-coil domain of TRAF6 and inhibit TRAF6 auto-ubiquitination and TRAF6-TAB2 association for the activation of NF-κB (Fig. 8, inhibition of TRAF6-TAB2 signaling axis). Simultaneously, ARRB2 can interrupt the interaction between TRAF6 and BECN1 and inhibit BECN1 ubiquitination for autophagy induction (Fig. 8, inhibition of TRAF6-BECN1 signaling axis). Consequently, ARRB2 can inhibit lung cancer progression regulated by two signaling axes, TRAF6-TAB2 signaling axis for NF-κB activation and the TRAF6-BECN1 signaling axis for autophagy induction, in response to TLR3 and TLR4 stimulation. Our current work might provide novel insights into the functional crosstalk between TLR-induced autophagy and lung cancer progression, thereby contributing to the development of a therapeutic strategy and/or a therapeutic target in the treatment of lung cancer.

## Supplementary information

Supplemental Material

Supplementary Table S1

Original Data File

checklist

## Data Availability

The data that support the findings of this study are available from the corresponding author upon reasonable request.
