# Supplementary material for: β‐arrestin 2 negatively regulates lung cancer progression by inhibiting the TRAF6 signaling axis for NF-κB activation and autophagy induced by TLR3 and TLR4
Source: Cell Death Dis. 2023 Jul 13;14(7):422. doi: 10.1038/s41419-023-05945-3 (PMC10344878; doi:10.1038/s41419-023-05945-3)
Supplement: Supplementary file 1 — Supplemental Material [file 41419_2023_5945_MOESM1_ESM.docx]

**Supplement Figure Legends**


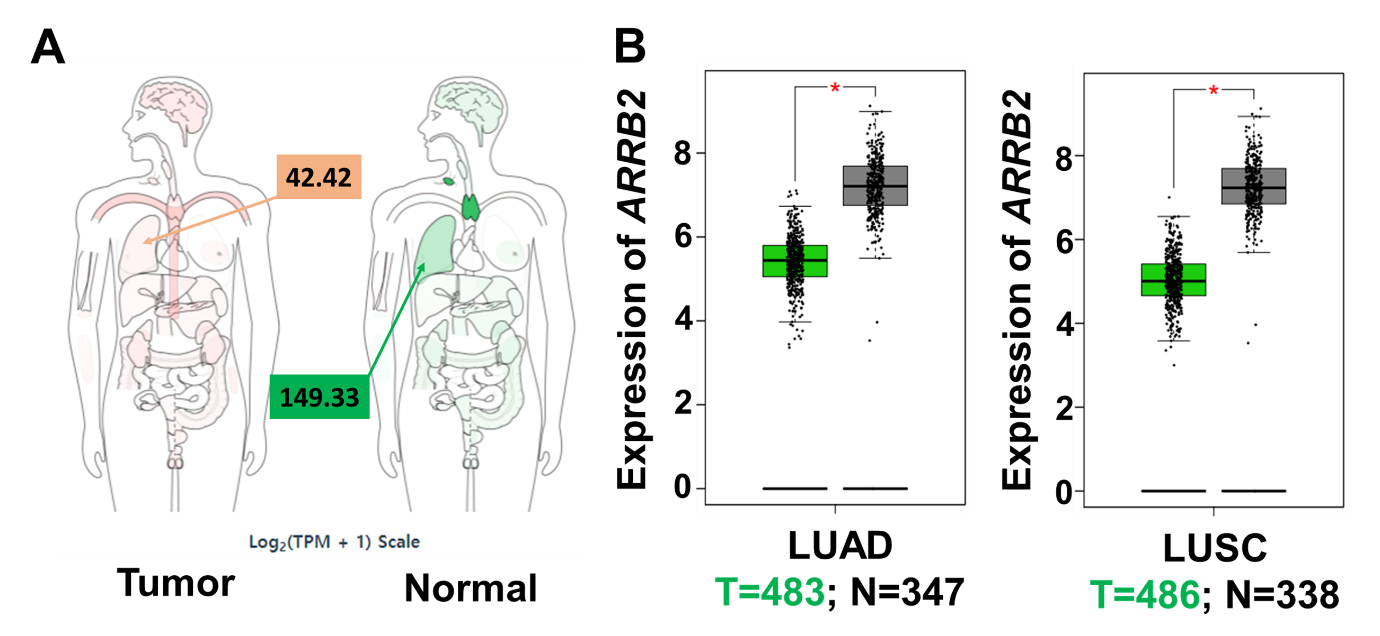


**Supplementary Fig. S1.** **ARRB2 expression in LUAD (Lung adenocarcinoma) and LUSC (Lung squamous cell carcinoma)**. **A** and **B**) ARRB2 expression was analyzed with data from The Cancer Genome Atlas through Gene expression Profiling Interactive Analysis (GEPIA, [http://gepia.cancer-pku.cn/detail.php?gene=ARRB2###](http://gepia.cancer-pku.cn/detail.php?gene=ARRB2)). Interactive bodymap was represented (**A**). ARRB2 expression on box plots was showed in LUAD and LUSC (**B**: left, LUAD; right, LUSC). * *p* < 0.05.


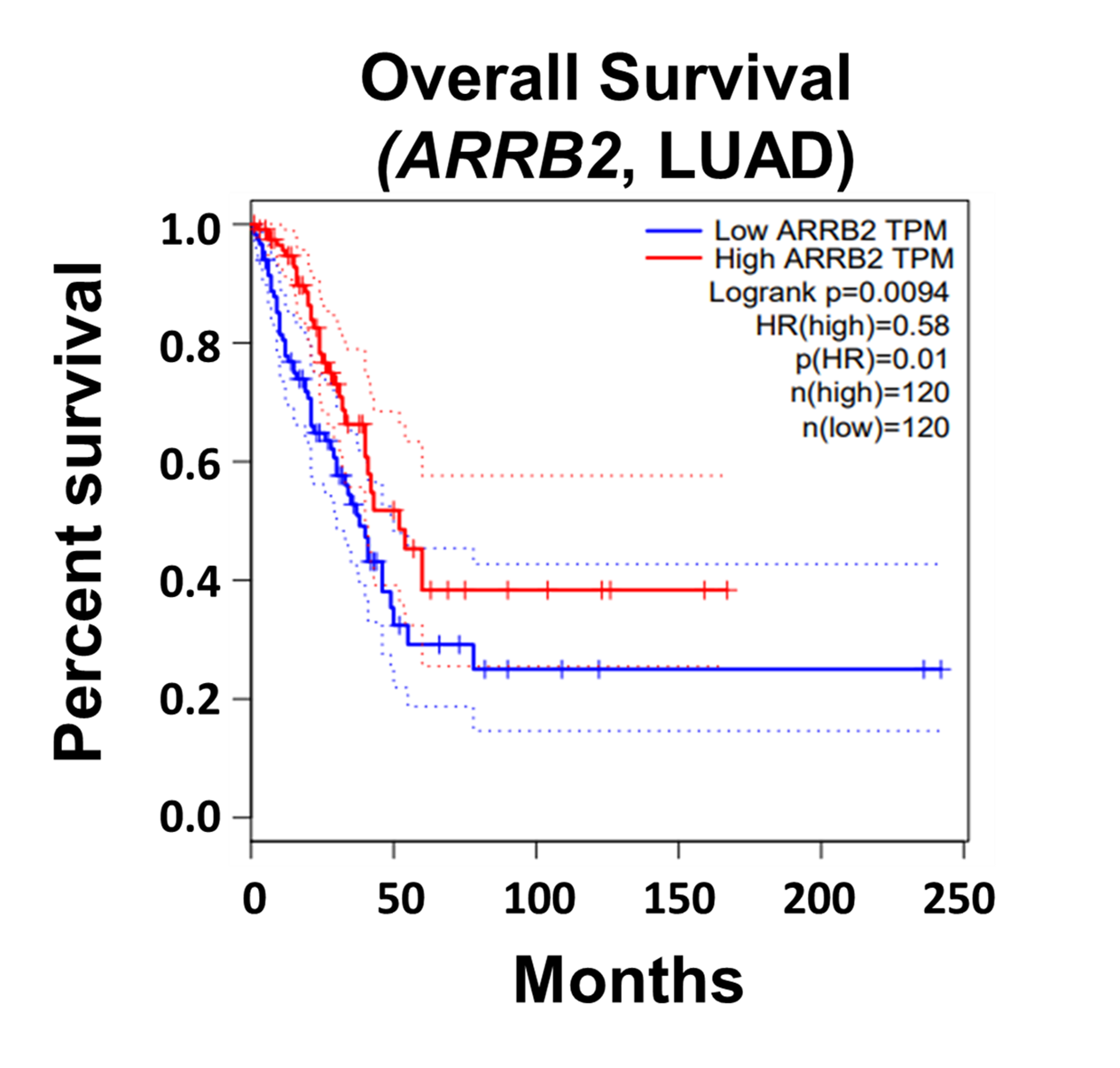


**Supplementary Fig. S2.** **Overall survival of LUAD patients**. Overall survival data was obtained from The Cancer Genome Atlas through Gene expression Profiling Interactive Analysis (GEPIA, [http://gepia.cancer-pku.cn/detail.php?gene=ARRB2###](http://gepia.cancer-pku.cn/detail.php?gene=ARRB2)). *p*=0.0094, low ARRB2 vs. high ARRB2.


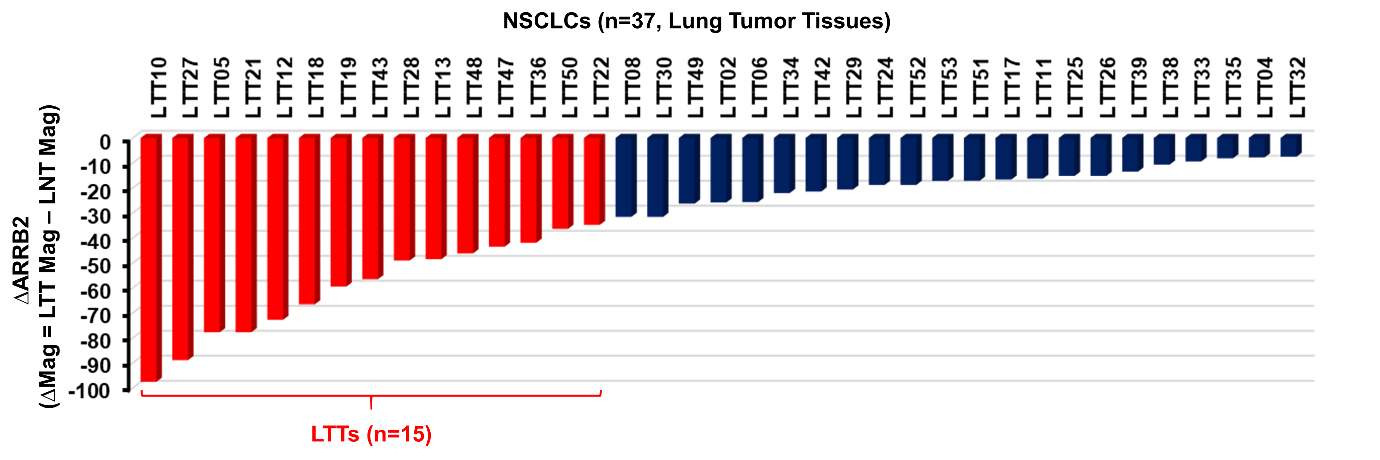


**Supplementary Fig. S3**. **Differential magnitude of ARRB2 expression between lung tumor tissues (LTTs) and matched lung normal tissues (LNTs) of NSCLC patients (n=37)**. Magnitude difference of ARRB2 (∆ARRB2) was obtained from the pre-processed microarray data between LTTs (n=37) and matched LNTs (n=37, ∆Mag = LTT Mag – LNT Mag). Top 15 LTTs with the downregulated ARRB2 were selected, as indicated in red boxes.


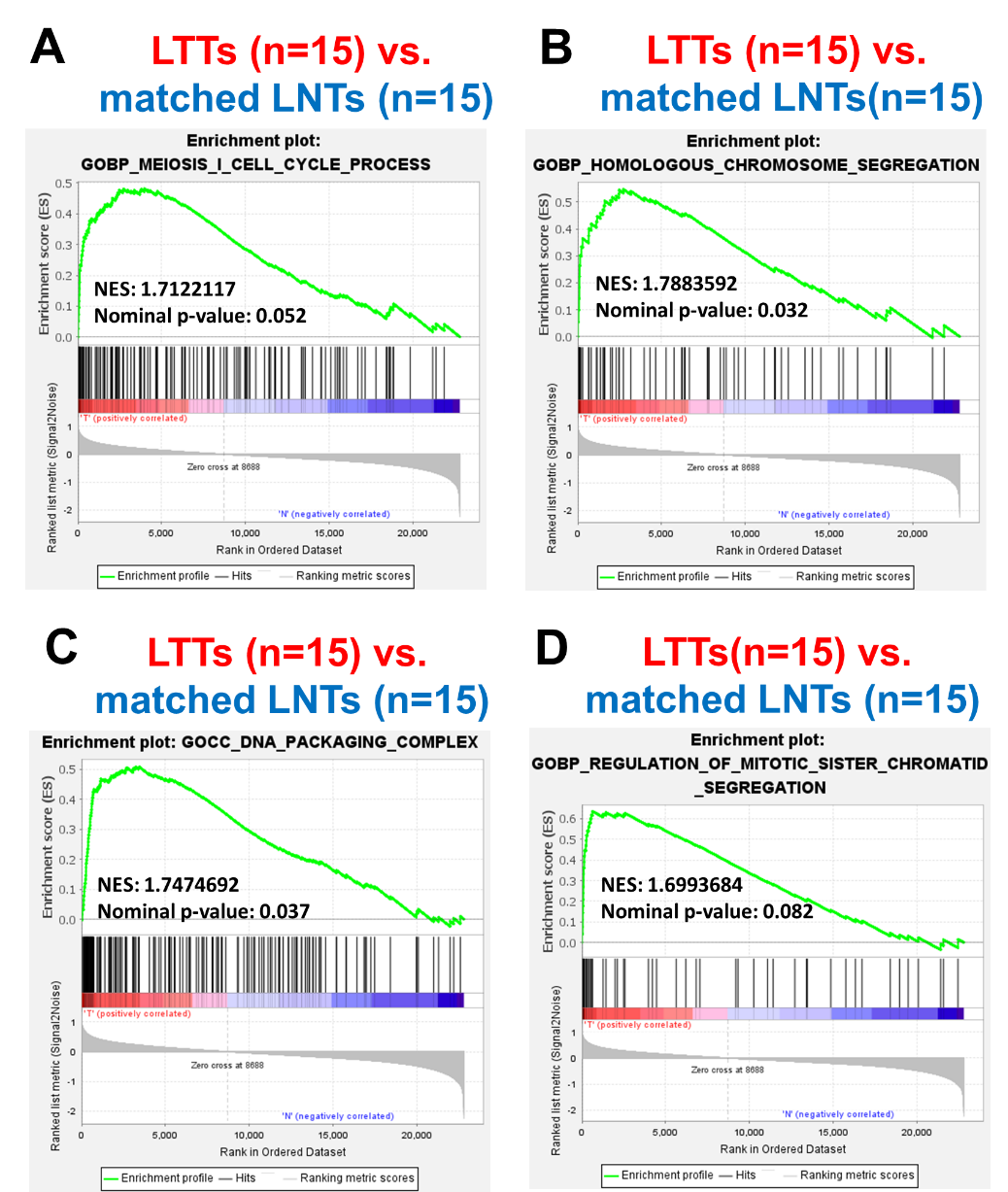


**Supplementary Fig. S4**. **Gene Set Enrichment Analysis (GSEA) between 15 LTTs and matched 15 LNTs of NSCLC patients.** **A-D.** According to the different magnitude of ARRB2 in LTTs vs. matched LNTs (Fig. S3), top 15 LTTs with the down-regulated ARRB2 in 37 LTTs were selected, and GSEA (http://www.gsea-msigdb.org/gsea/index.jsp) was performed in 15 LTTs vs. matched 15 LNTs. Four gene sets, MEIOSIS I CELL CYCLE PROCESS (**A**), HOMOLOGOUS CHROMOSOME SEGREGATION (**B**), DNA PACKAGING COMPLEX (**C**) and REGULATION OF MITOTIC SISTER CHROMATID SEGREGATION (**D**), were represented. NES and Nominal p-value were indicated in each inner panel.


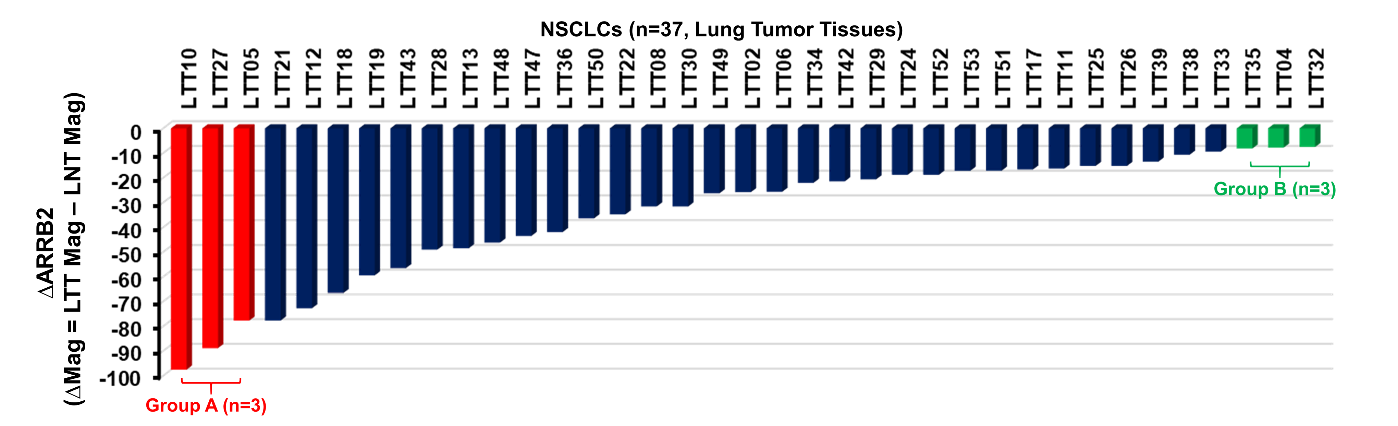


**Supplementary Fig. S5**. **Differential magnitude of ARRB2 expression between lung tumor tissues (LTTs) and matched lung normal tissues (LNTs) of NSCLC patients (n=37)**. Magnitude difference of ARRB2 (∆ARRB2) was obtained from the pre-processed microarray data between LTTs (n=37) and matched LNTs (n=37, ∆Mag = LTT Mag – LNT Mag). 6 LTTs were selected from the top (3 LTTs with highly down-regulated ARRB2, Group A, red boxes) and bottom (3 LTTs with lowly down-regulated ARRB2, Group B, green boxes).


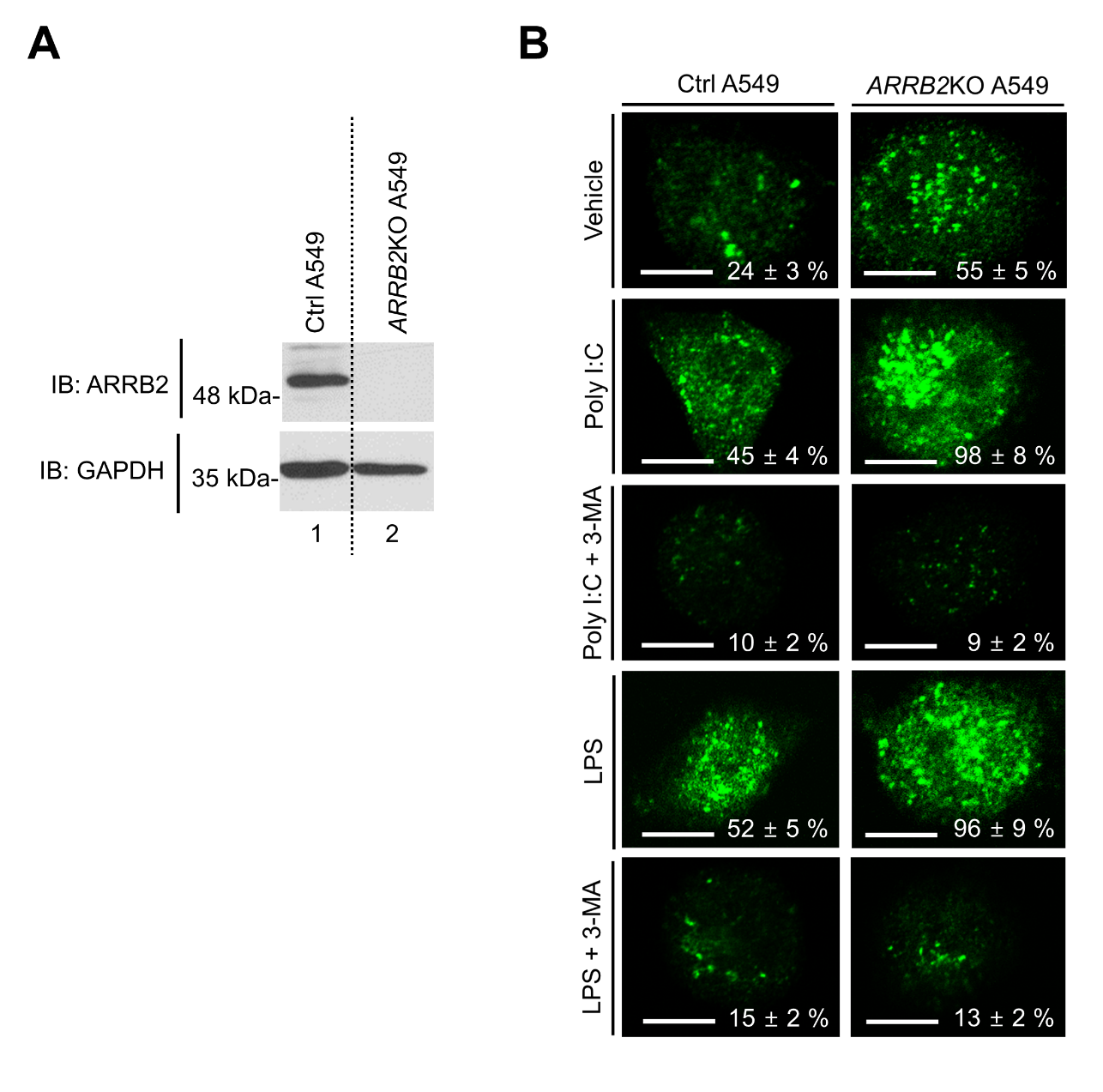


**Supplementary Fig. S6**. **Generation of ARRB2-knockout (*ARRB2*KO) A549 cells and LC3 puncta assay**. **A.** ARRB2-knockout (*ARRB2*KO) A549 cells were generated by CRISPR/Cas9 gene-editing method, as described in Material and Methods. ARRB2 expression was verified by anti-ARRB2 antibody, along with anti-GAPDH as a loading control. **B.** Ctrl A549 and *ARRB2*KO A549 cells were treated with vehicle (DMSO, 0.1% v/v concentration), Poly I:C (5 μg/ml), or LPS (15 μg/ml) in the presence or absence of 3-MA (5 mM) for 6 h, and immunolabeled with LC3 antibody, as described in Material and Methods. Images shown are representative fluorescence confocal microscopic photographs. Quantification of the percentage of cells with autophagosomes is shown (± SD, n = 50 cells). Scale bar: 10 μm.


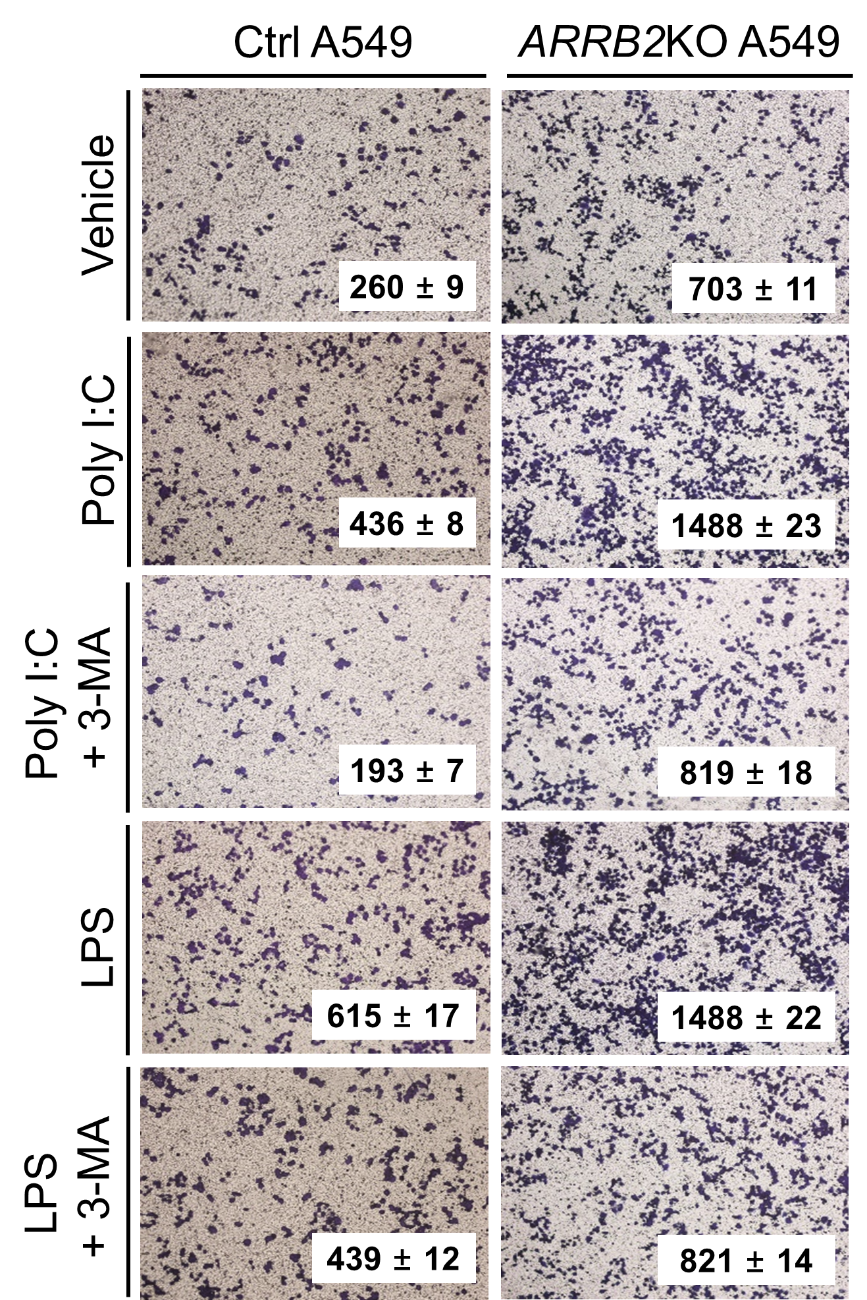


**Supplementary Fig. S7**. ***ARRB2*KO A549 cells exhibit enhancements of cell invasion induced by TLR3 and TLR4.** Ctrl A549 and *ARRB2*KO A549 cells treated with vehicle (DMSO, 0.1% v/v concentration), Poly I:C (15 μg/ml), or LPS (15 μg/ml) in the presence or absence of 3-MA (5 mM) for 24 h, as described in Material and Methods. The cells were counted. The results are presented as the mean ± SD of three independent experiments.

**
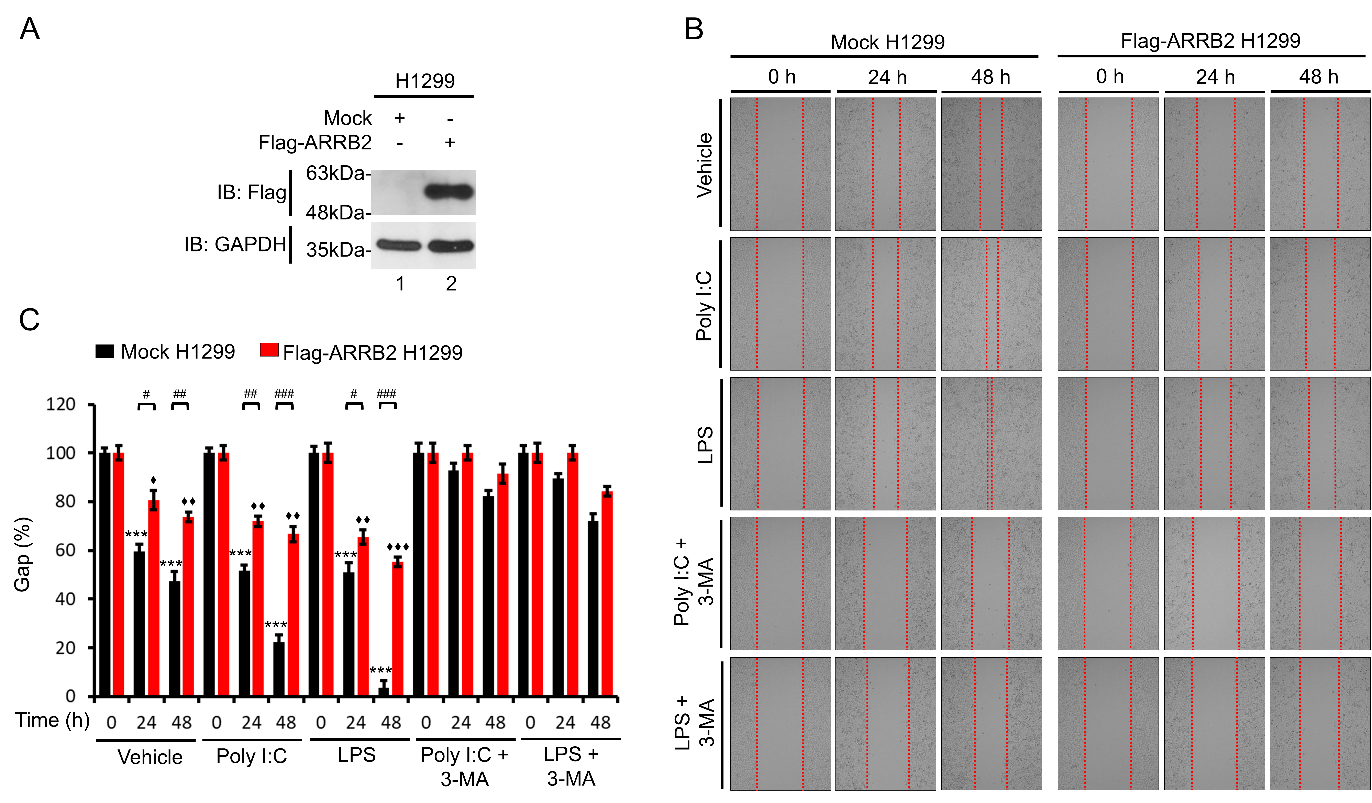
**

**Supplementary Fig. S8**. **ARRB2-overexperssed H1299 cells exhibit attenuations of cell migration induced by TLR3 and TLR4. A.** H1299 cells were transfected with Mock as a control vector or with Flag-ARRB2. Cell lysates were immunoblotted with an anti-Flag antibody and anti-GAPDH (as a loading control). **B** and **C**. Mock H1299 and Flag-ARRB2 H1299 cells were treated with vehicle (DMSO, 0.1% v/v concentration), Poly I:C (15 μg/ml), or LPS (10 μg/ml) in the presence or absence of 3-MA (3 mM) for different time periods (**B**). The residual gap between migrating cells from the opposing wound edge is expressed as a percentage of the initial scraped area (± SD, n = 3 different plates) (**C**). ^#^ *p* < 0.05, ^##^ *p* < 0.01, and ^###^ *p* < 0.001; *** *p* < 0.001, 0 h of vehicle, Poly I:C, and LPS vs. 24 h or 48 h of vehicle, Poly I:C, and LPS in mock H1299; ^♦^ *p* < 0.05, ^♦♦^ *p* < 0.01, and ^♦♦♦^ *p* < 0.001, 0 h of vehicle, Poly I:C, and LPS vs. 24 h or 48 h of vehicle, Poly I:C, and LPS in Flag-ARRB2 H1299.

**
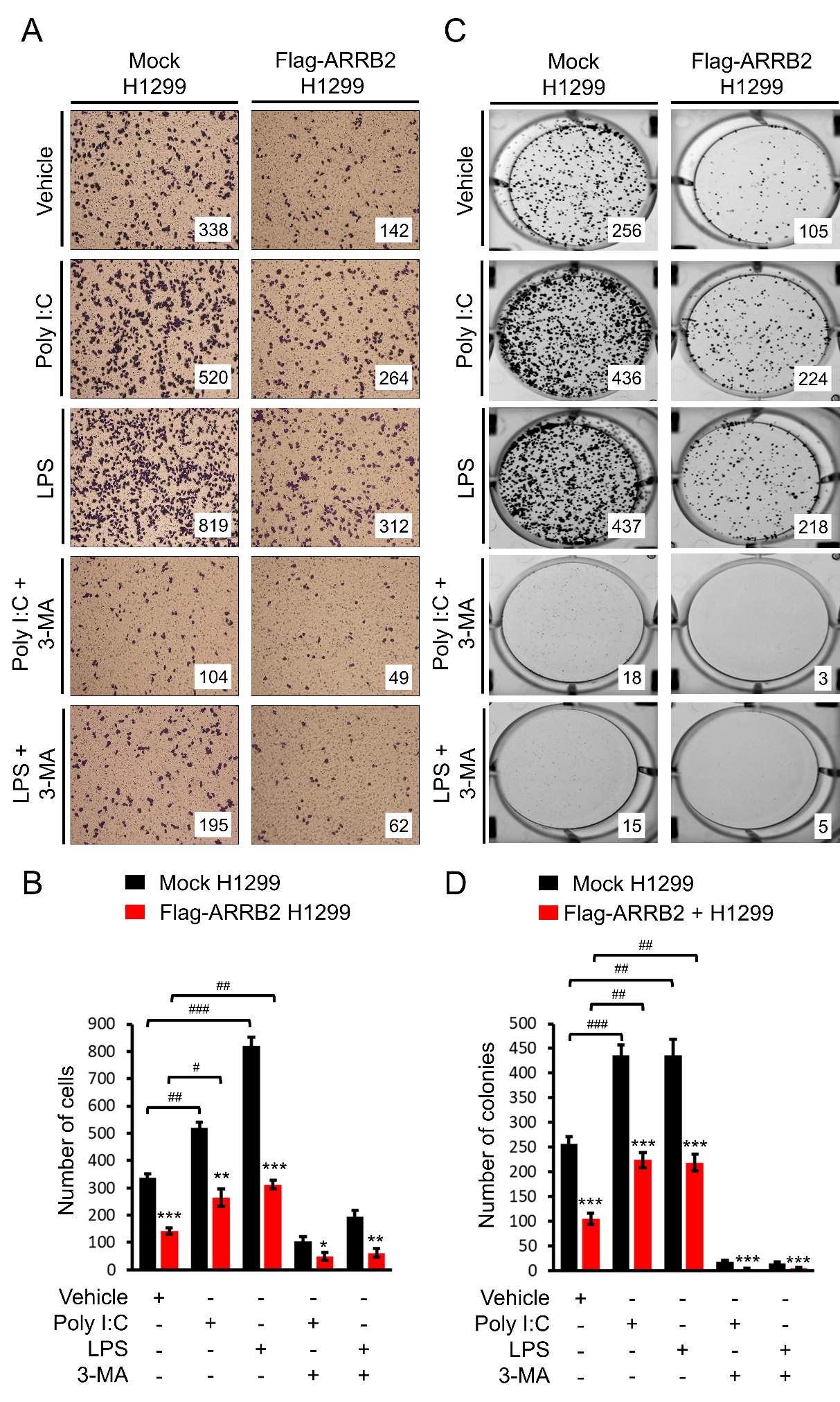
**

**Supplementary Fig. S9**. **ARRB2-overexperssed H1299 cells exhibit attenuations of cell invasion and colony formation induced by TLR3 and TLR4. A** and **B.** Mock H1299 and Flag-ARRB2 H1299 cells were treated with vehicle (DMSO, 0.1% v/v concentration), Poly I:C (25 μg/ml), or LPS (15 μg/ml) in the presence or absence of 3-MA (7 mM) (**A**). The number of migrating cells were counted. Results are presented as the mean ± SD of three independent experiments (**B**). ^#^ *p* < 0.05, ^##^ *p* < 0.01, and ^###^ *p* < 0.001; * *p* < 0.05, ** *p* < 0.01, and *** *p* < 0.001, vehicle, Poly I:C, LPS, Poly I:C plus 3-MA, or LPS plus 3-MA in Mock H1299 vs. vehicle, Poly I:C, LPS, Poly I:C plus 3-MA, or LPS plus 3-MA in Flag-ARRB2 H1299. **C** and **D**. Mock H1299 and Flag-ARRB2 H1299 cells were treated with vehicle (DMSO, 0.1% v/v concentration), Poly I:C (5 μg/ml), or LPS (5 μg/ml) in the presence or absence of 3-MA (4 mM) (**C**). The number of colonies was measured using Adobe Photoshop software, and results are presented as mean ± SD of three independent experiments (**D**, n = 3 plates). ^#^ *p* < 0.05, ^##^ *p* < 0.01, and ^###^ *p* < 0.001; *** *p* < 0.001, vehicle, Poly I:C, LPS, Poly I:C plus 3-MA, or LPS plus 3-MA in Mock H1299 vs. vehicle, Poly I:C, LPS, Poly I:C plus 3-MA, or LPS plus 3-MA in Flag-ARRB2 H1299.

**
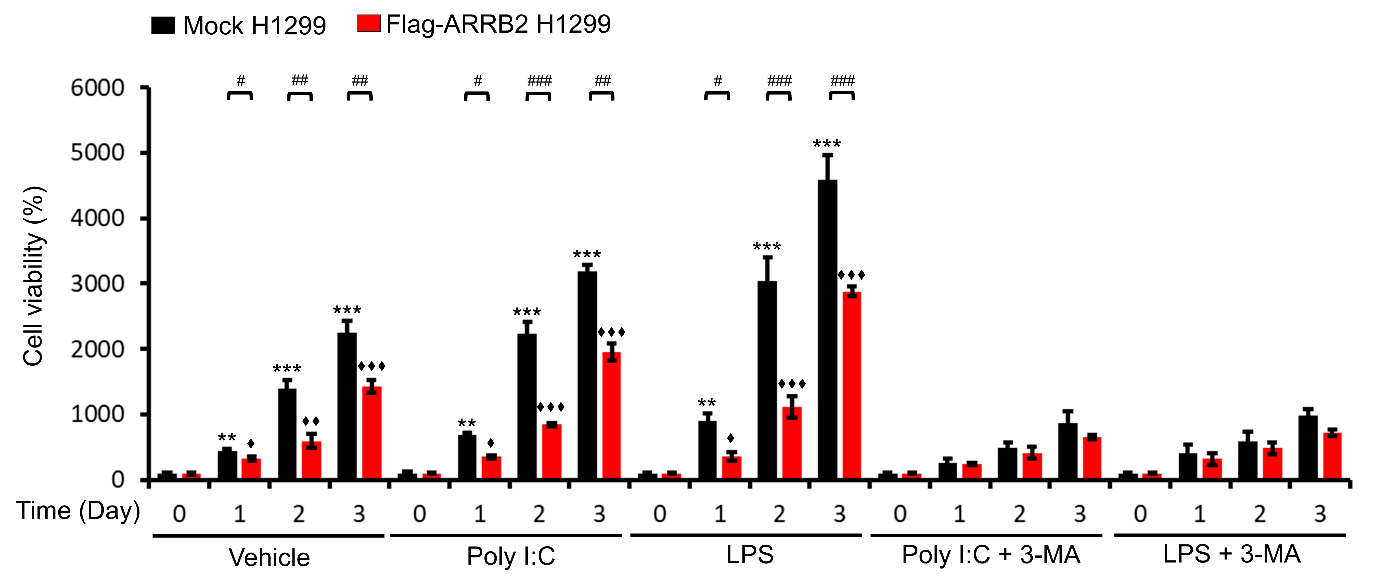
**

**Supplementary Fig. S10**. **ARRB2-overexperssed H1299 cells exhibit attenuations of cell proliferation induced by TLR3 and TLR4.** Mock H1299 and Flag-ARRB2 H1299 cells were treated with vehicle (DMSO, 0.1% v/v concentration), Poly I:C (5 μg/ml), or LPS (5 μg/ml) in the presence or absence of 3-MA (2 mM). MTT assay was then performed, as described in in Material and Methods. Results are presented as the mean ± SD of three independent experiments. ^#^ *p* < 0.05, ^##^ *p* < 0.01, and ^###^ *p* < 0.001; ** *p* < 0.01 *** *p* < 0.001, 0 day of vehicle, Poly I:C, and LPS vs. 2 day or 3 day of vehicle, Poly I:C, and LPS in mock H1299; ^♦^ *p* < 0.05, ^♦♦^ *p* < 0.01, and ^♦♦♦^ *p* < 0.001, 0 day of vehicle, Poly I:C, and LPS vs. 2 day or 3 day of vehicle, Poly I:C, and LPS in Flag-ARRB2 H1299.

**Supplement Table information**

**Supplementary Table S1**. Magnitude difference of ARRB2 (∆ARRB2) between lung tumor tissues (LTTs) and matched lung normal tissues (LNTs) of NSCLC patients (n=37)
