# Supplementary material for: β‐arrestin 2 negatively regulates lung cancer progression by inhibiting the TRAF6 signaling axis for NF-κB activation and autophagy induced by TLR3 and TLR4
Source: Cell Death Dis. 2023 Jul 13;14(7):422. doi: 10.1038/s41419-023-05945-3 (PMC10344878; doi:10.1038/s41419-023-05945-3)
Supplement: Supplementary file 2 — Supplementary Table S1 [file 41419_2023_5945_MOESM2_ESM.pdf]

Supplementary Table S1. Magnitude difference of ARRB2 (ΔARRB2) between lung tumor tissues (LTTs) and matched lung normal tissues (LNTs) of NSCLC patients (n=37)

Lung tumor Tissues (LTTs)	Magnitude (Mag)	Matched Lung Normal Tissues (Matched LNTs)	Magnitude (Mag)	Magnitude difference of ARRB2 (ΔARRB2) (ΔMag = LTT Mag – LNT Mag)
LTT10.AVG_Signal	80.45263	LNT10.AVG_Signal	178.003505	-97.550875
LTT27.AVG_Signal	35.645019	LNT27.AVG_Signal	124.49723	-88.852211
LTT05.AVG_Signal	36.772385	LNT05.AVG_Signal	114.463435	-77.69105
LTT21.AVG_Signal	40.47714	LNT21.AVG_Signal	118.16808	-77.69094
LTT12.AVG_Signal	11.1829655	LNT12.AVG_Signal	83.924835	-72.7418695
LTT18.AVG_Signal	30.68437	LNT18.AVG_Signal	97.245825	-66.561455
LTT19.AVG_Signal	39.730845	LNT19.AVG_Signal	99.171035	-59.44019
LTT43.AVG_Signal	3.4702675	LNT43.AVG_Signal	59.934008	-56.4637405
LTT28.AVG_Signal	37.43957	LNT28.AVG_Signal	86.444965	-49.005395
LTT13.AVG_Signal	22.329957	LNT13.AVG_Signal	70.823175	-48.493218
LTT48.AVG_Signal	5.4634665	LNT48.AVG_Signal	51.643454	-46.1799875
LTT47.AVG_Signal	1.3568285	LNT47.AVG_Signal	44.858985	-43.5021565
LTT36.AVG_Signal	4.8448535	LNT36.AVG_Signal	46.7617275	-41.916874
LTT50.AVG_Signal	16.5295555	LNT50.AVG_Signal	52.90279	-36.3732345
LTT22.AVG_Signal	46.64946	LNT22.AVG_Signal	81.42554	-34.77608
LTT08.AVG_Signal	-0.7313829	LNT08.AVG_Signal	30.829214	-31.56059685
LTT30.AVG_Signal	16.8853235	LNT30.AVG_Signal	48.44332	-31.5579965
LTT49.AVG_Signal	8.537901	LNT49.AVG_Signal	34.758902	-26.221001
LTT02.AVG_Signal	7.8737465	LNT02.AVG_Signal	33.684405	-25.8106585
LTT06.AVG_Signal	15.786971	LNT06.AVG_Signal	41.42942925	-25.64245825
LTT34.AVG_Signal	26.176216	LNT34.AVG_Signal	48.2031965	-22.0269805
LTT42.AVG_Signal	3.3540045	LNT42.AVG_Signal	24.7511725	-21.397168
LTT29.AVG_Signal	10.5986675	LNT29.AVG_Signal	31.1960084	-20.5973409
LTT24.AVG_Signal	30.6412345	LNT24.AVG_Signal	49.44575	-18.8045155
LTT52.AVG_Signal	1.7273735	LNT52.AVG_Signal	20.4949475	-18.767574
LTT53.AVG_Signal	23.9283305	LNT53.AVG_Signal	41.097693	-17.1693625
LTT51.AVG_Signal	0.5221185	LNT51.AVG_Signal	17.635195	-17.1130765
LTT17.AVG_Signal	12.4702965	LNT17.AVG_Signal	29.067912	-16.5976155
LTT11.AVG_Signal	16.7151	LNT11.AVG_Signal	32.974115	-16.259015
LTT25.AVG_Signal	39.6582325	LNT25.AVG_Signal	54.85255	-15.1943175
LTT26.AVG_Signal	-3.282738	LNT26.AVG_Signal	11.889483	-15.172221
LTT39.AVG_Signal	6.904955	LNT39.AVG_Signal	20.3756925	-13.4707375
LTT38.AVG_Signal	17.2070195	LNT38.AVG_Signal	27.85008	-10.6430605
LTT33.AVG_Signal	14.6009825	LNT33.AVG_Signal	23.98483495	-9.38385245
LTT35.AVG_Signal	31.309315	LNT35.AVG_Signal	39.44426	-8.134945
LTT04.AVG_Signal	36.72251	LNT04.AVG_Signal	44.490259	-7.767749
LTT32.AVG_Signal	21.4487395	LNT32.AVG_Signal	28.9783275	-7.529588
