# Supplementary material for: β‐arrestin 2 negatively regulates lung cancer progression by inhibiting the TRAF6 signaling axis for NF-κB activation and autophagy induced by TLR3 and TLR4
Source: Cell Death Dis. 2023 Jul 13;14(7):422. doi: 10.1038/s41419-023-05945-3 (PMC10344878; doi:10.1038/s41419-023-05945-3)

Figure 3A

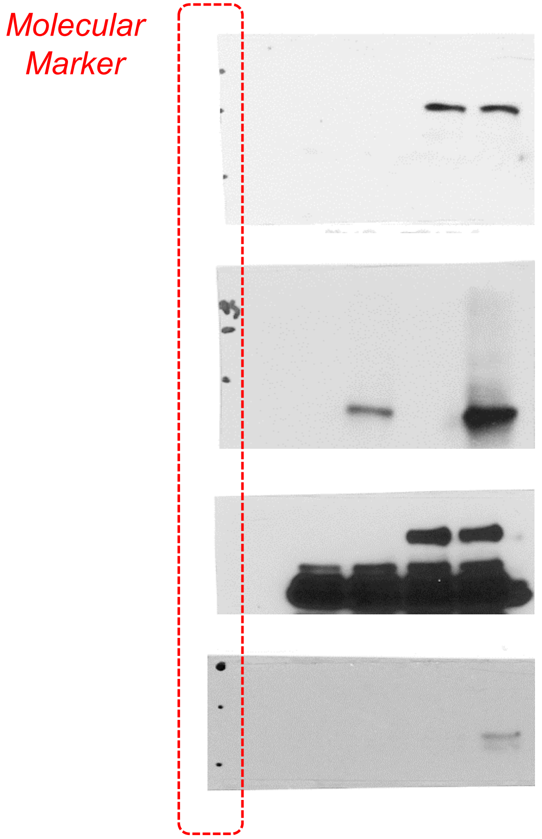

Figure 3C

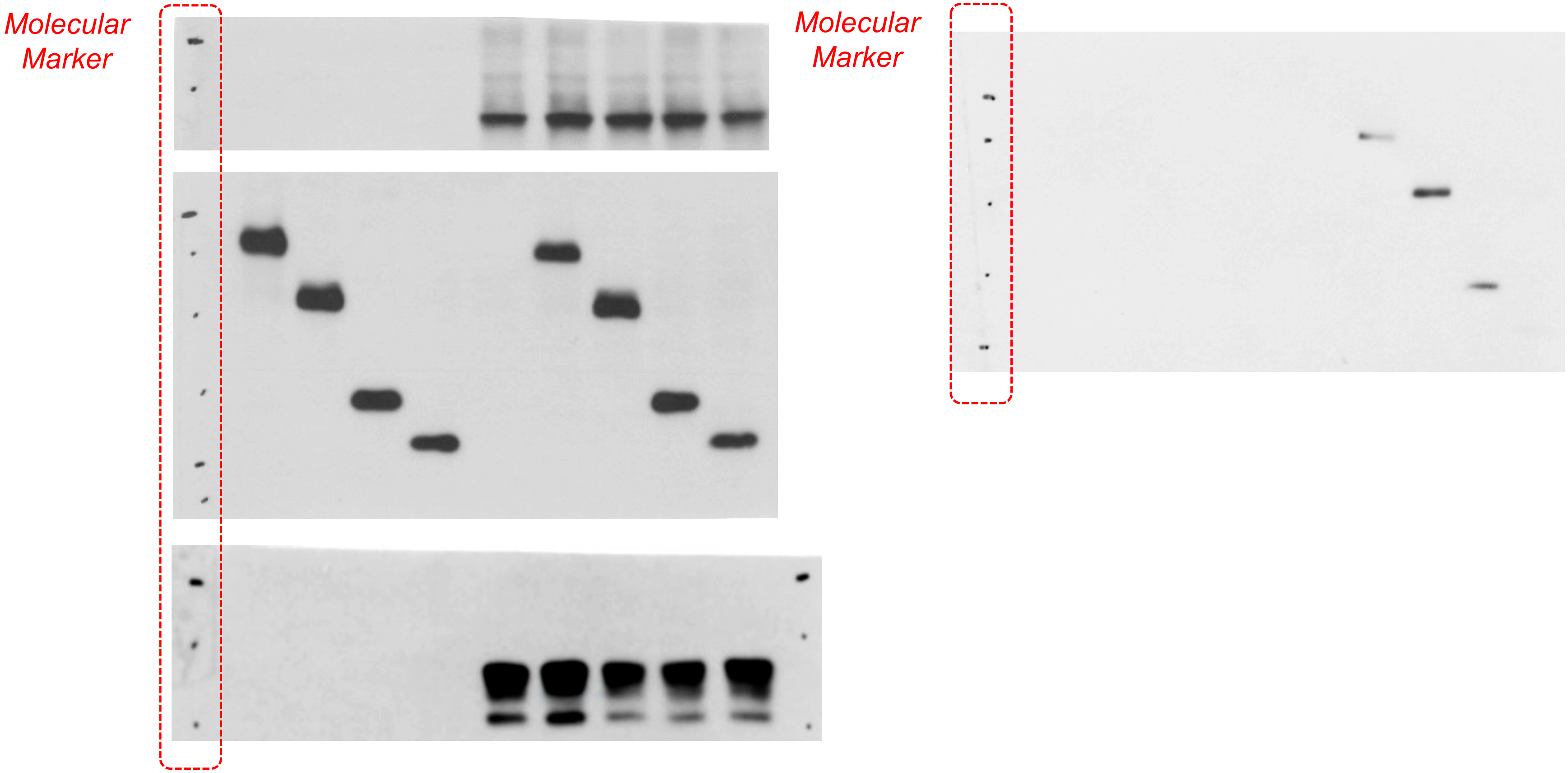

Figure 3E

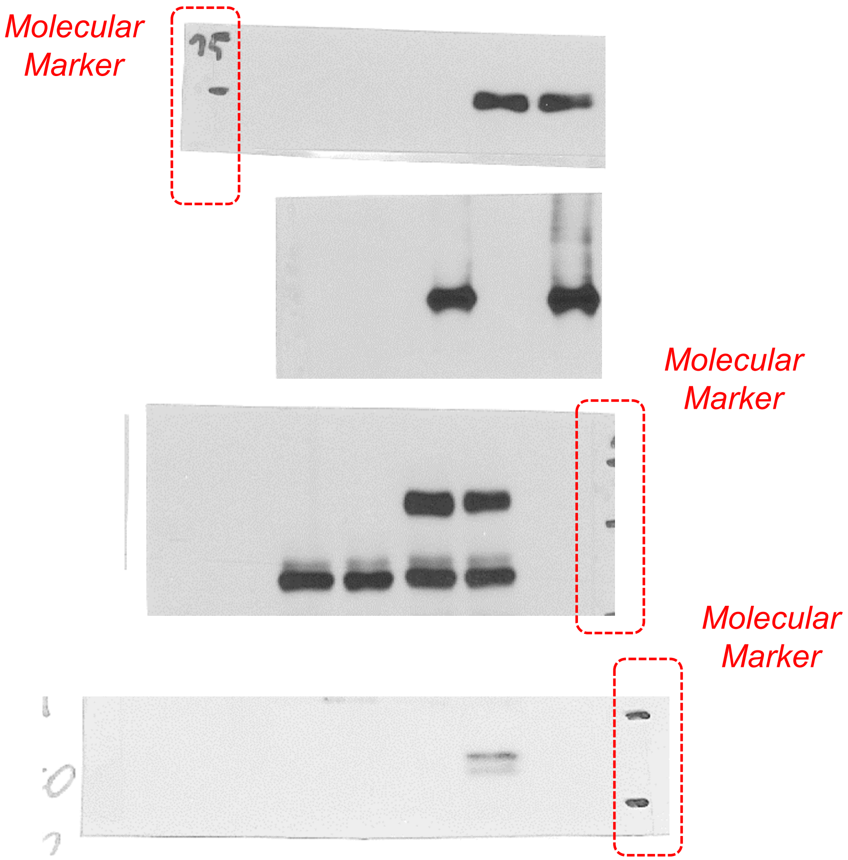

Figure 3G

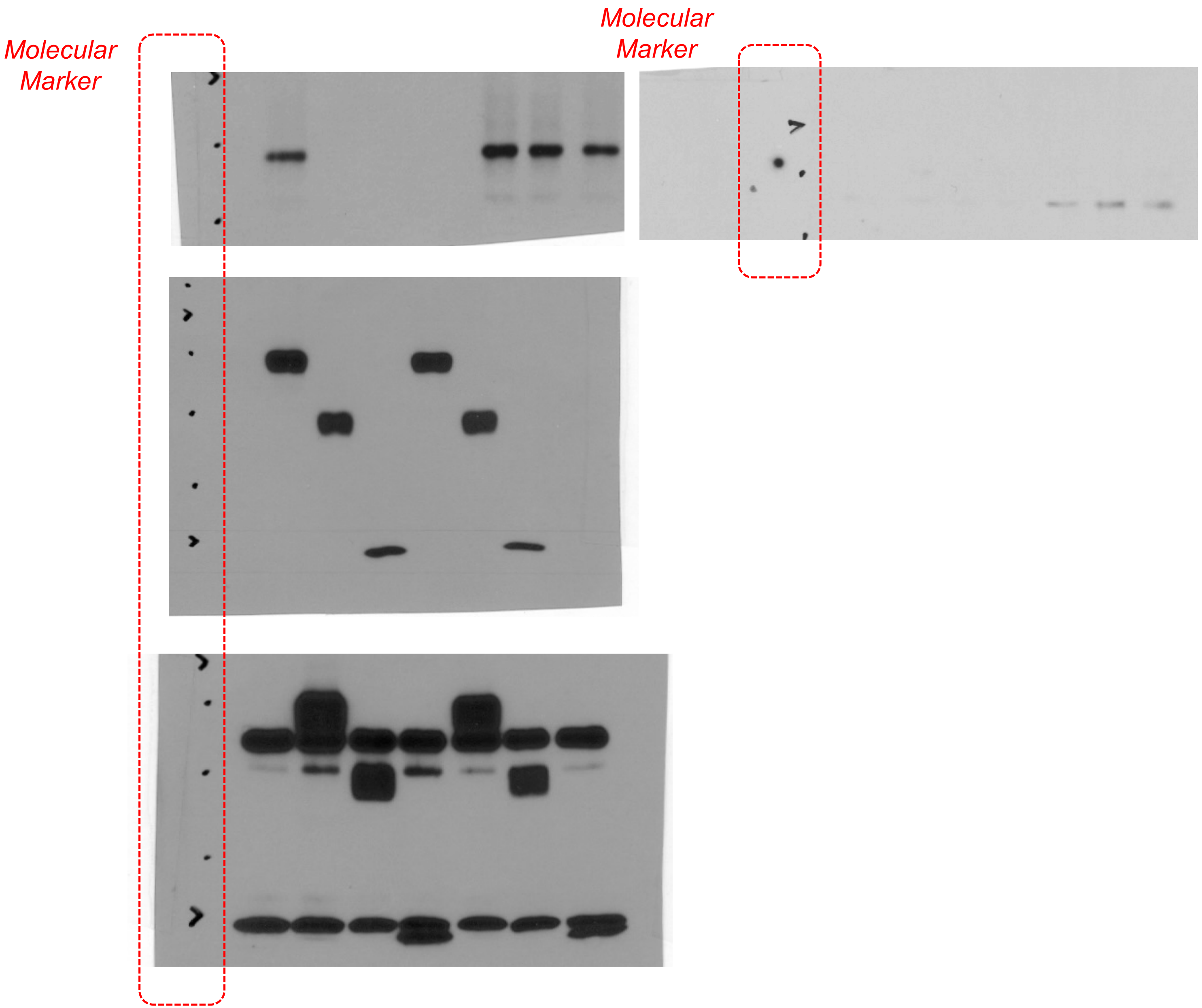

Figure 4A

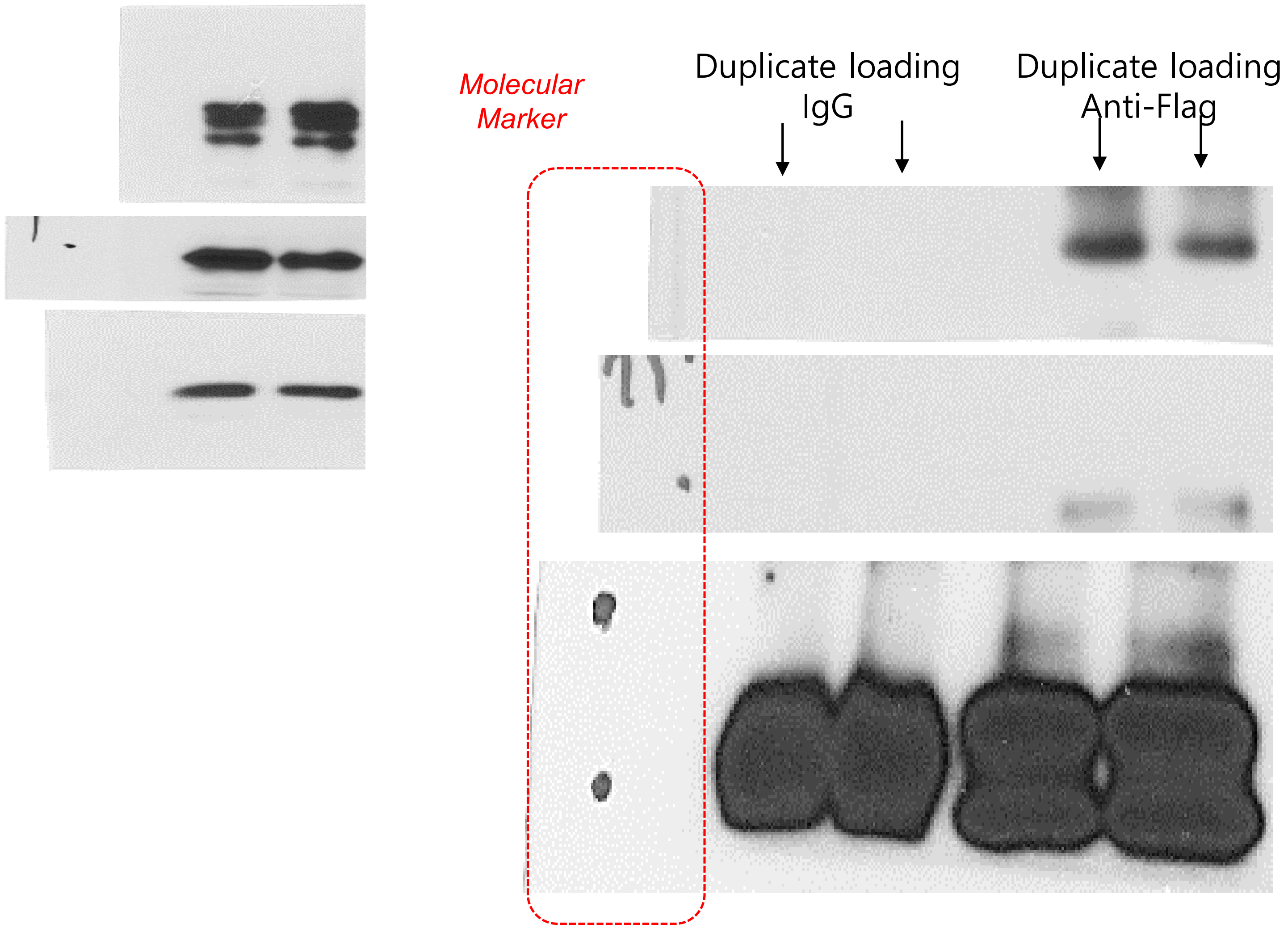

Molecular Marker
Duplicate loading IgG
Duplicate loading Anti-Flag

Figure 4B

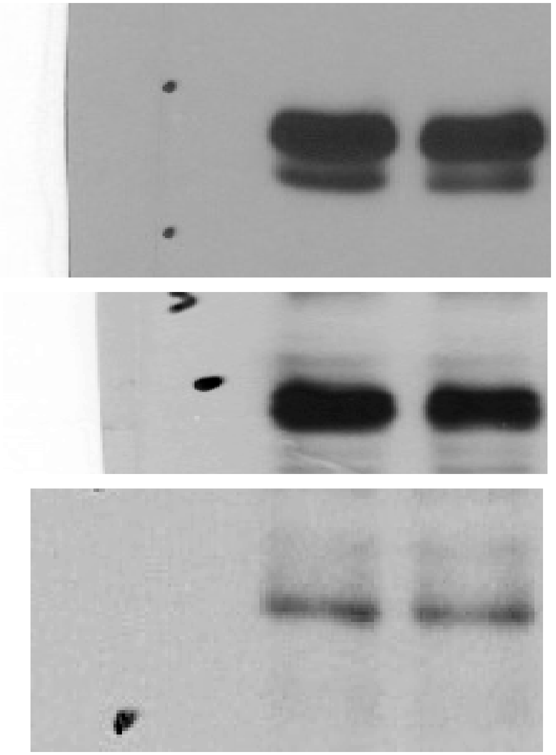

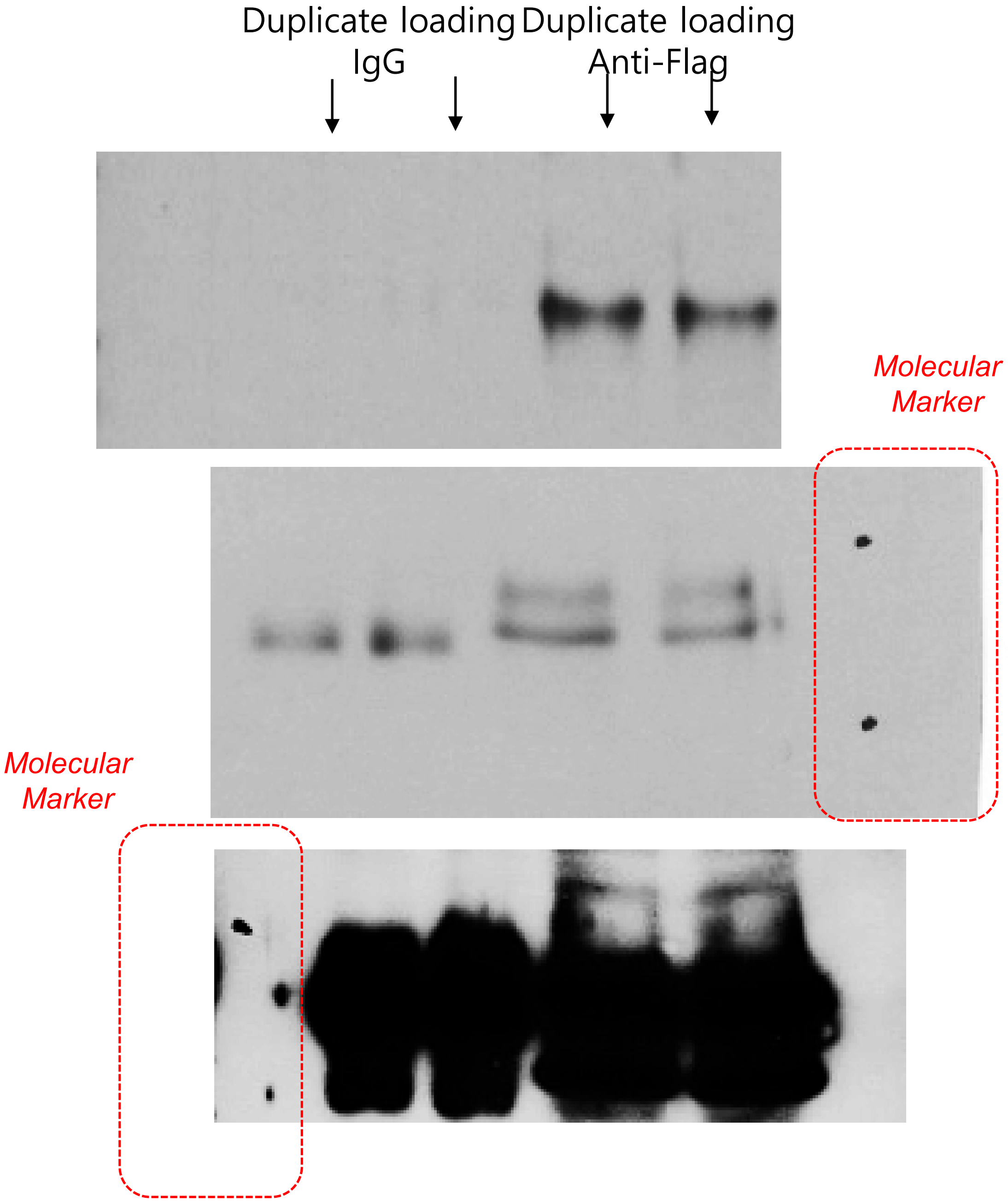
Duplicate loading
IgG
Duplicate loading
Anti-Flag
Molecular Marker
Molecular Marker

Figure 4C

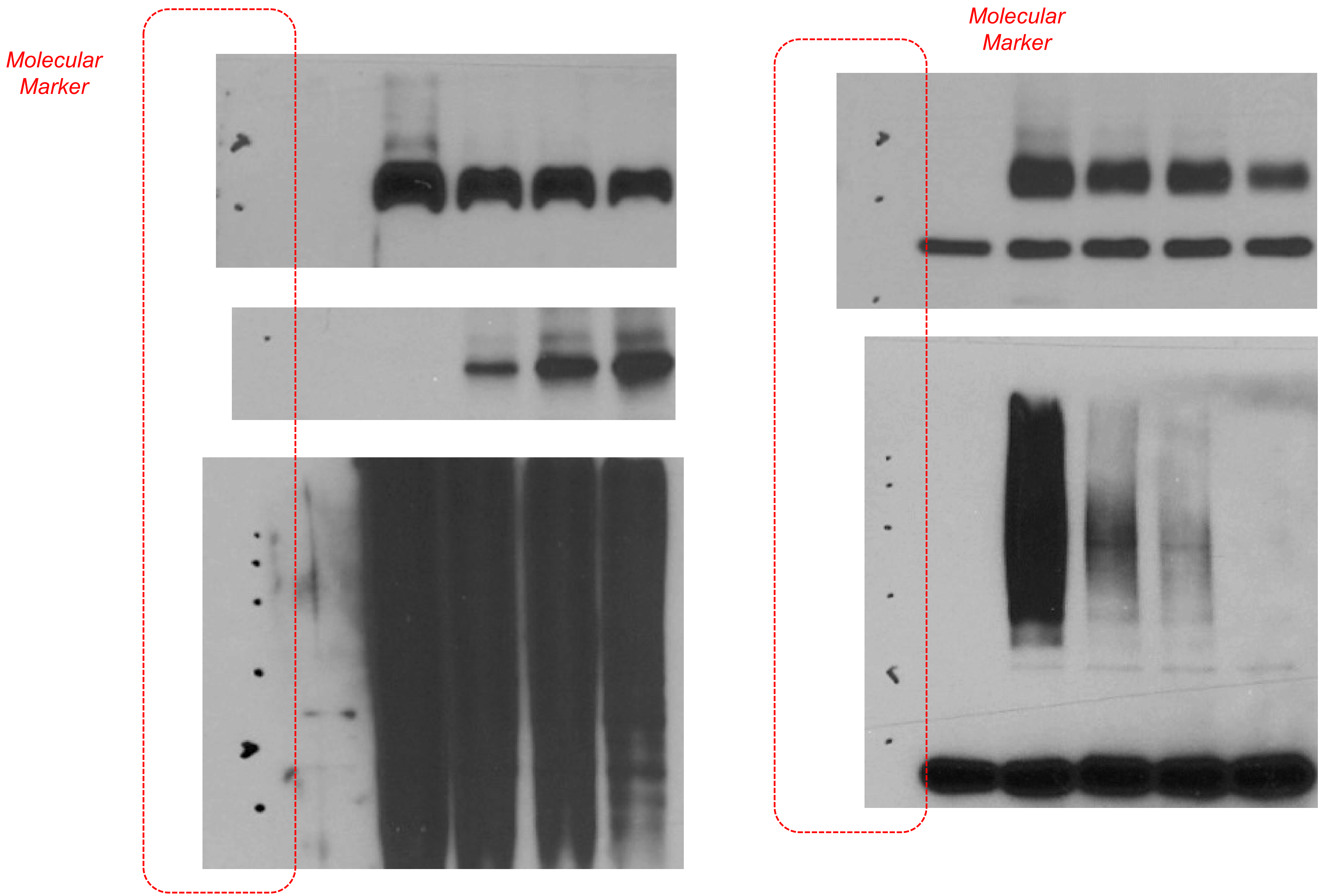

Figure 4D

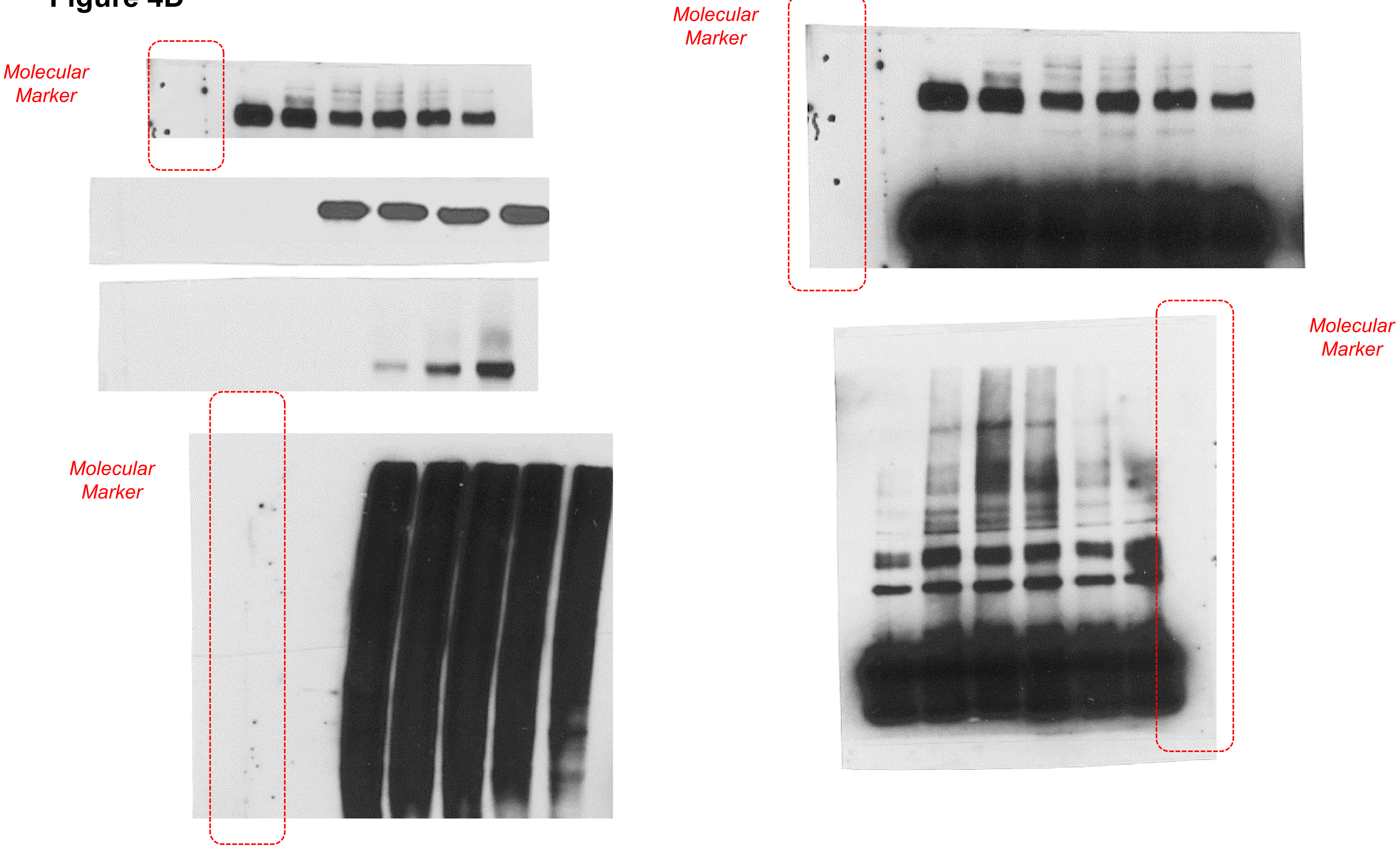

Molecular Marker
Molecular Marker
Molecular Marker
Molecular Marker

Figure 4E

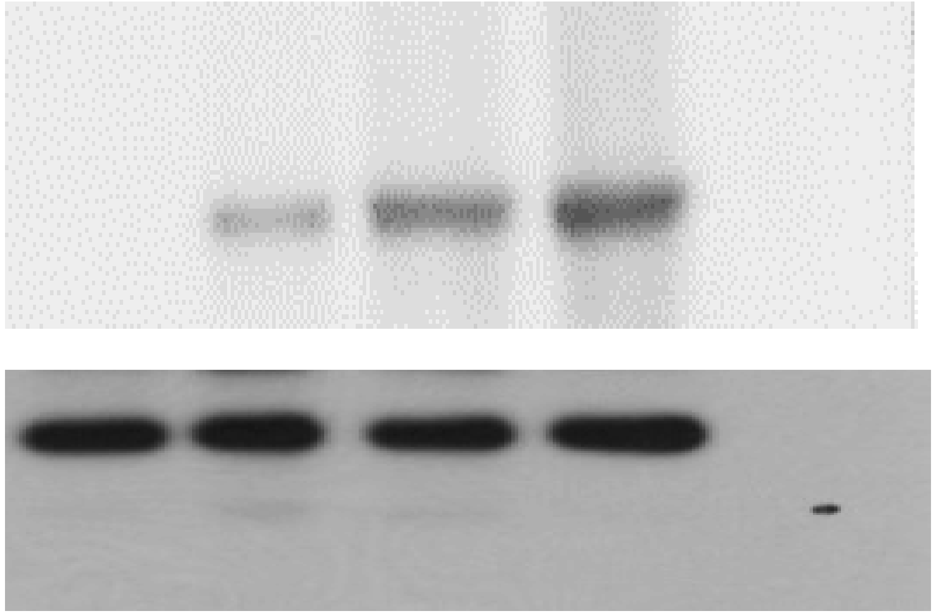

Figure 4G

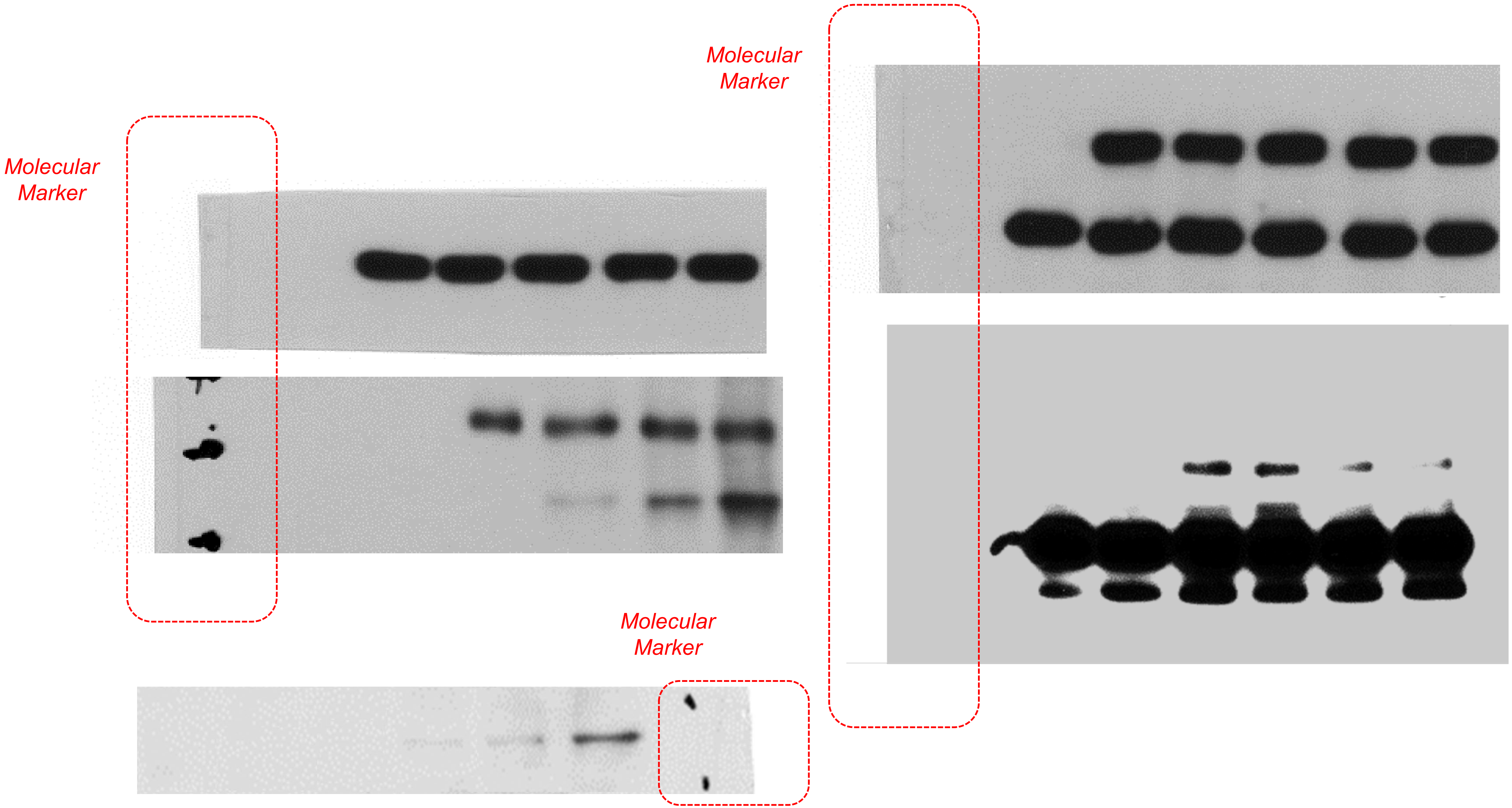

Figure 4I

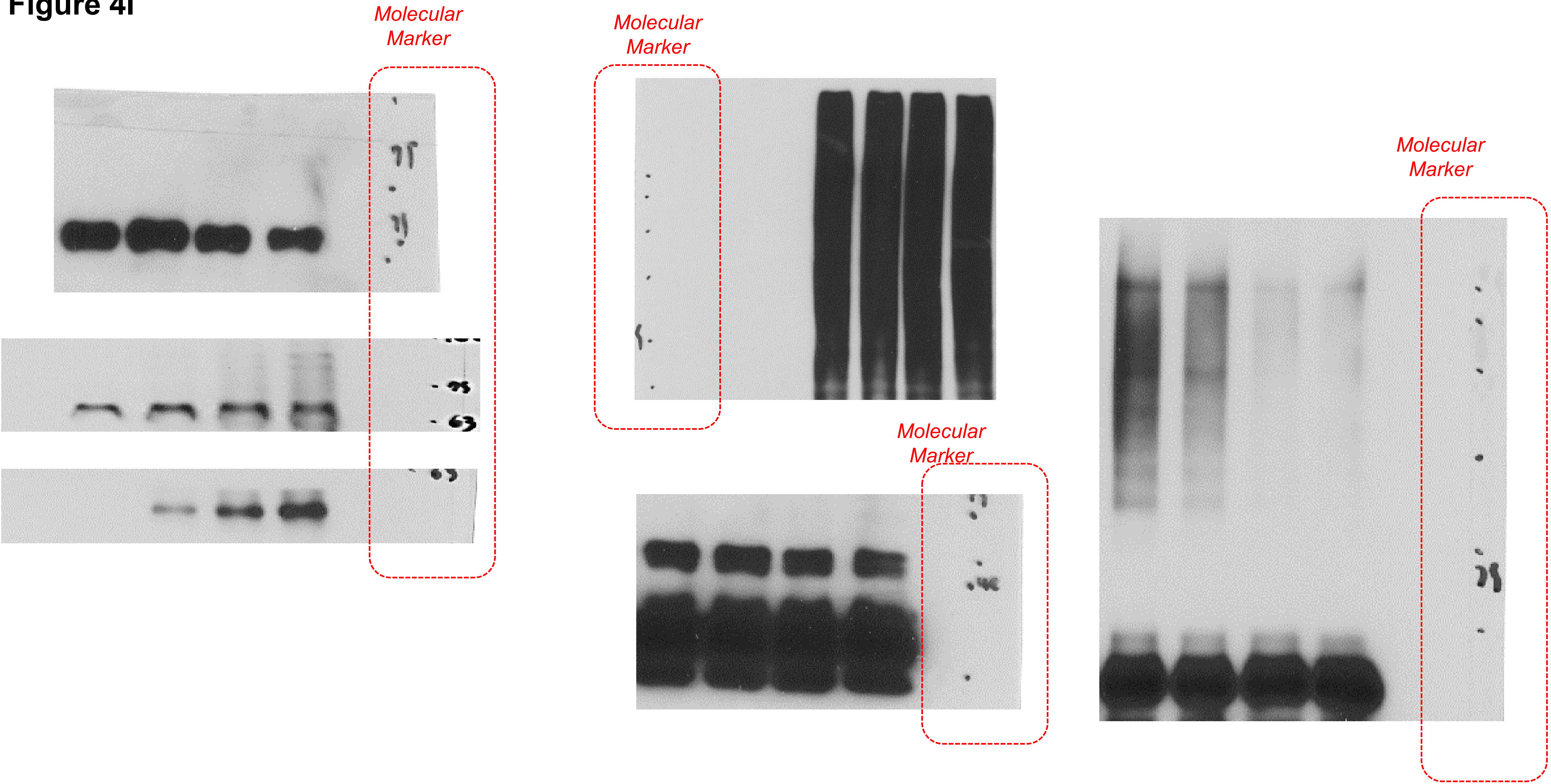
Molecular Marker
Molecular Marker
Molecular Marker
Molecular Marker

Figure 5A

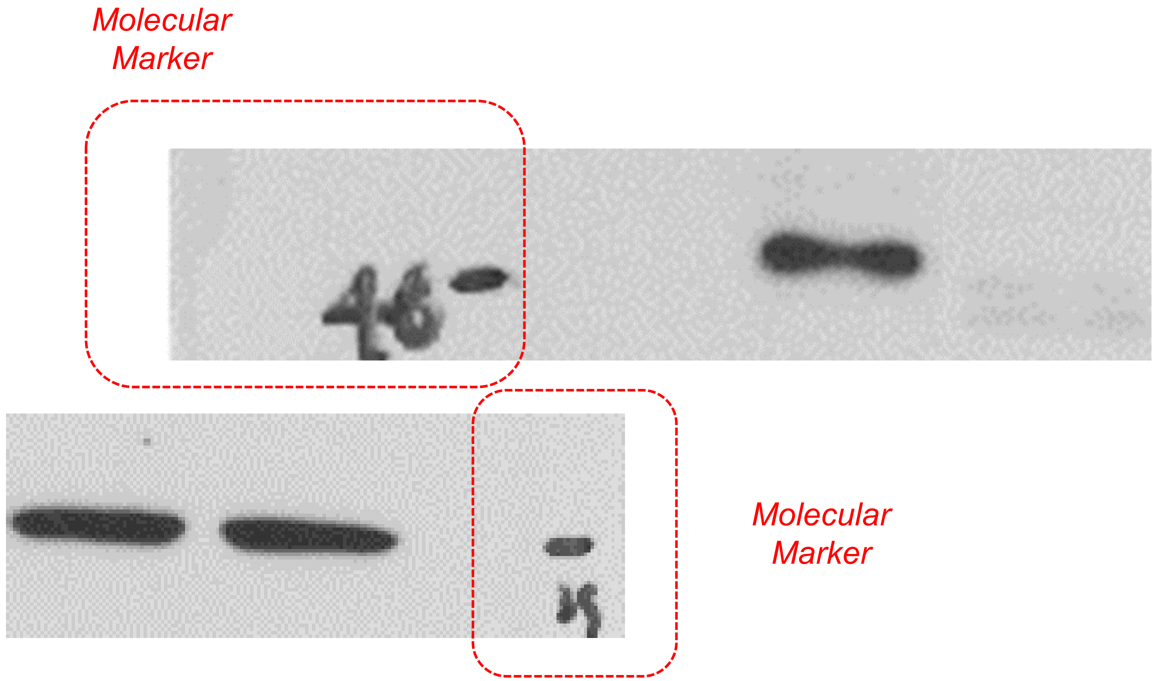

Figure 5B

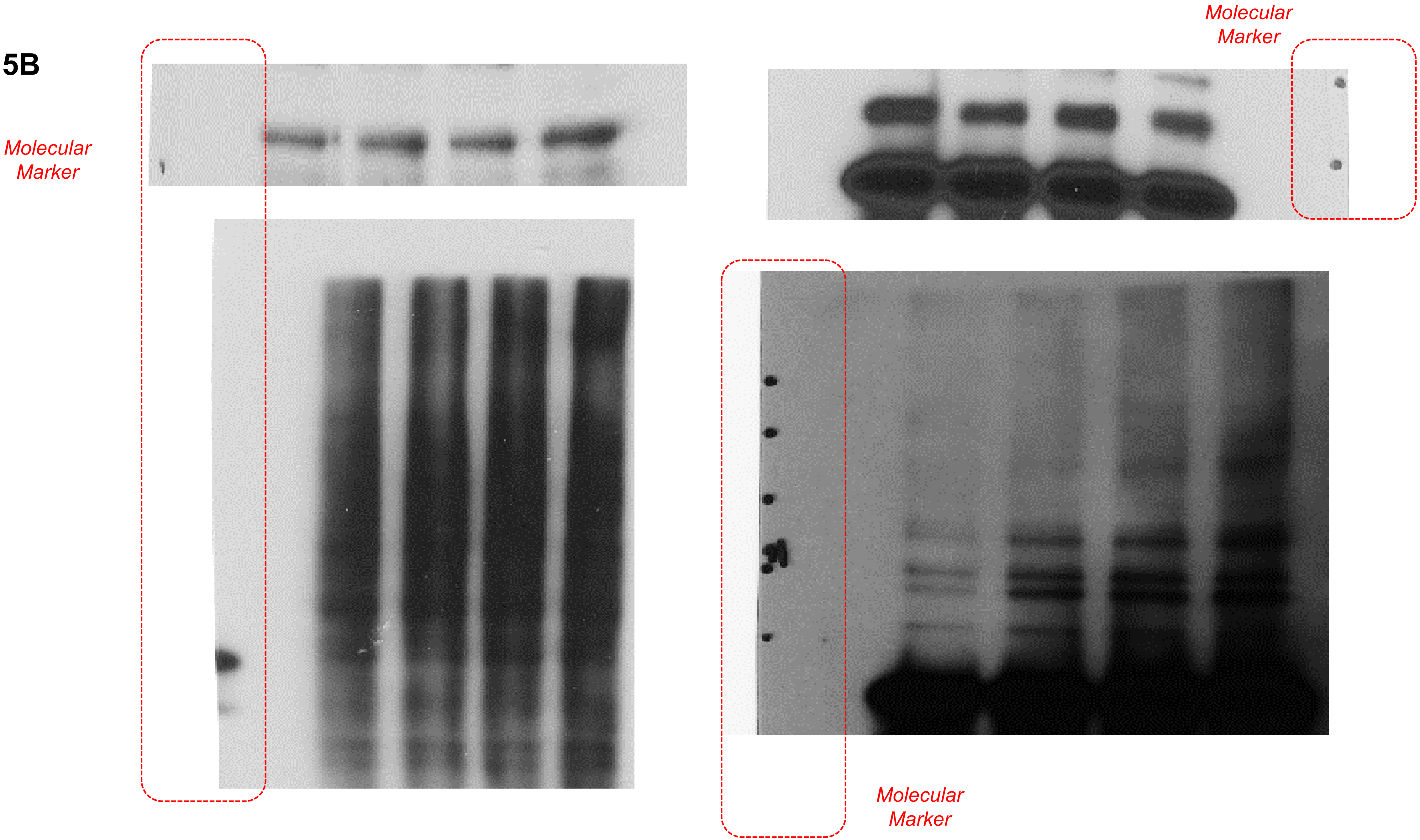

Figure 5D

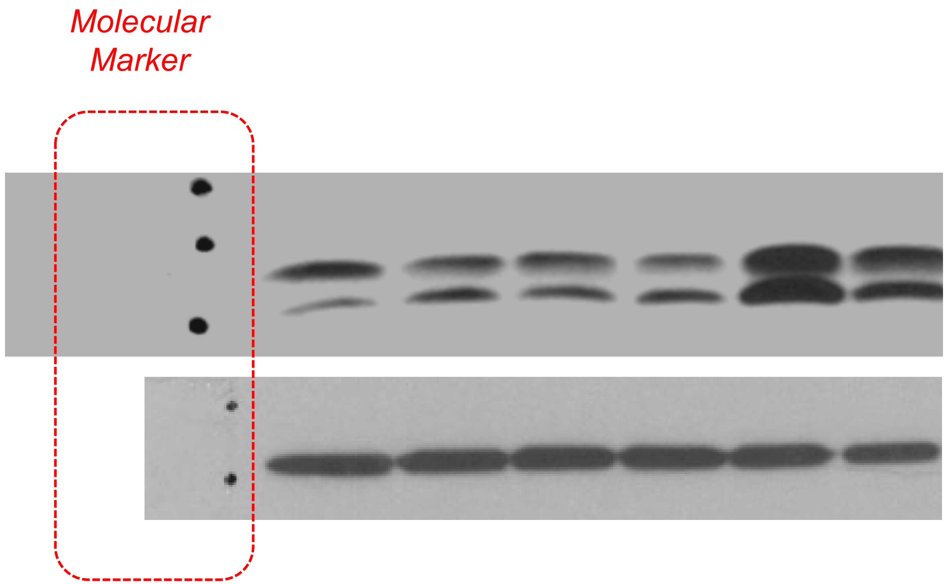

Figure 5C

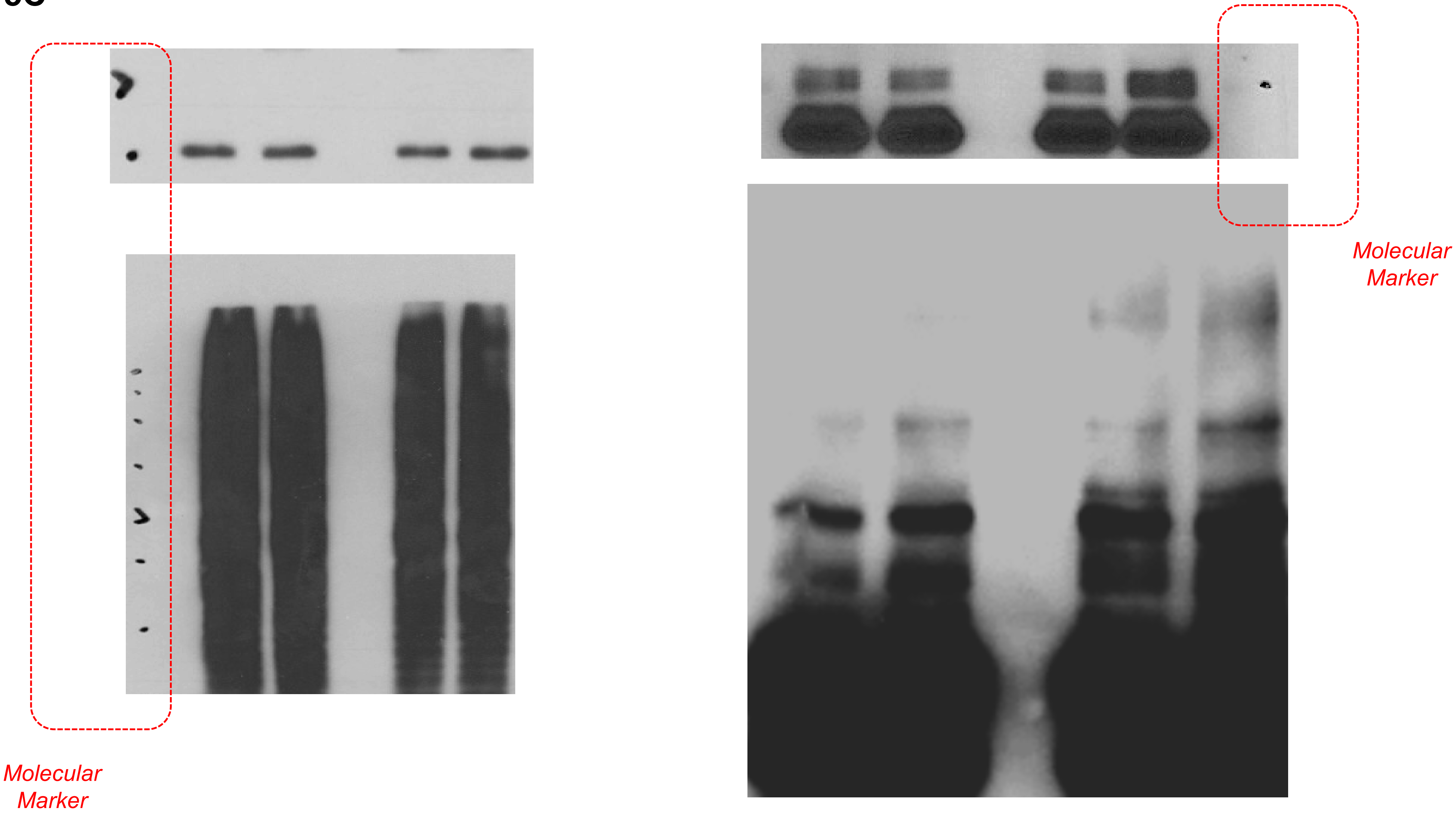

Figure S6A

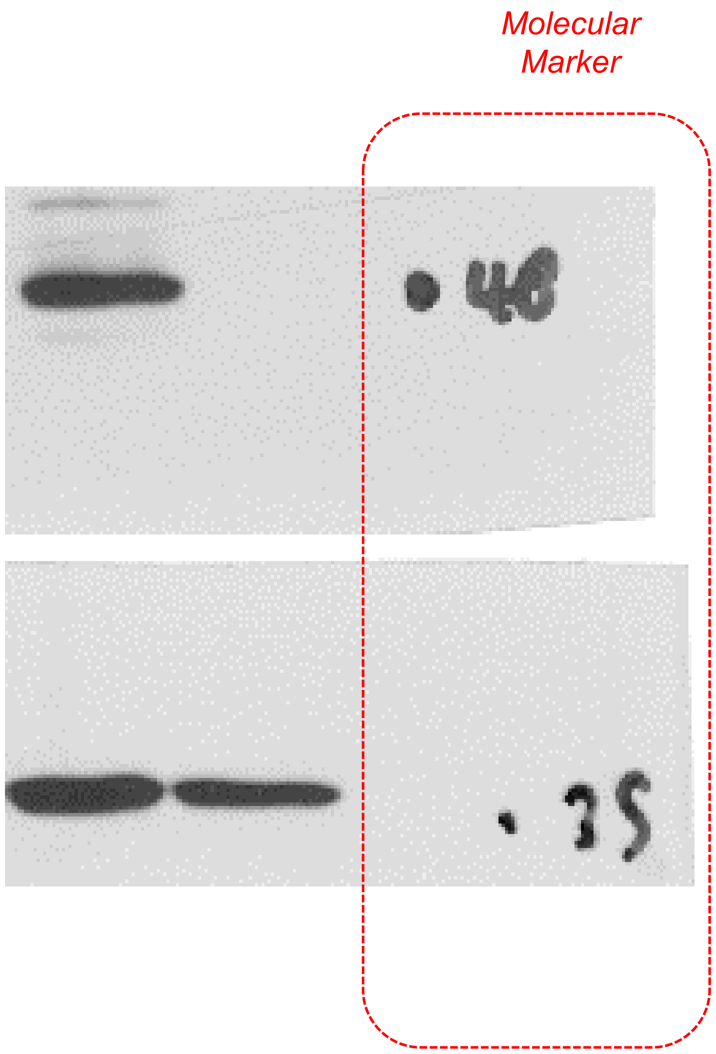

Figure S8A

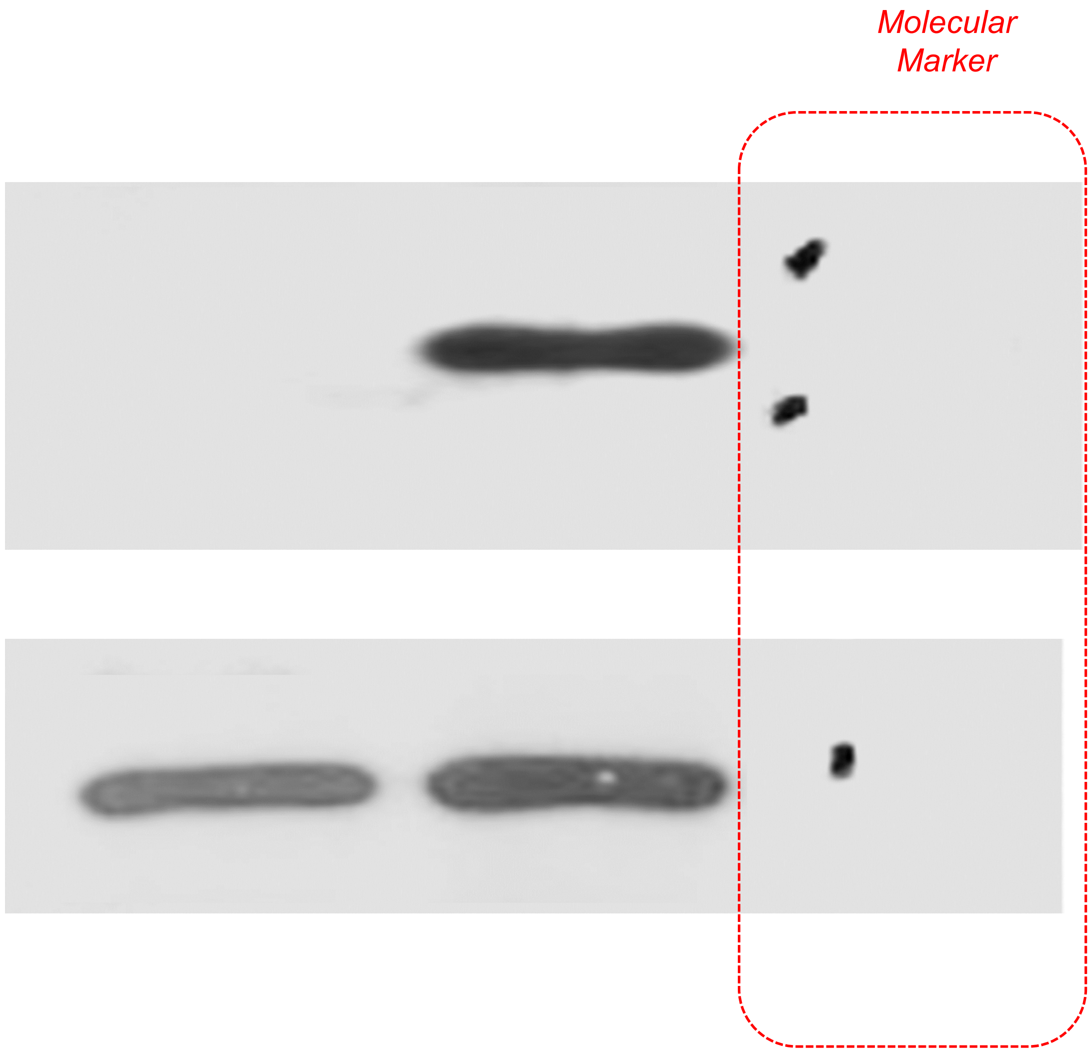

Supplement: Supplementary file 3 — Original Data File [file 41419_2023_5945_MOESM3_ESM.pdf]
